# The Immune System in Children with Malnutrition—A Systematic Review

**DOI:** 10.1371/journal.pone.0105017

**Published:** 2014-08-25

**Authors:** Maren Johanne Heilskov Rytter, Lilian Kolte, André Briend, Henrik Friis, Vibeke Brix Christensen

**Affiliations:** 1 Department of Nutrition, Exercise and Sports, Faculty of Science, University of Copenhagen, Frederiksberg, Denmark; 2 Department of Infectious Diseases, Copenhagen University Hospital, Hvidovre, Denmark; 3 Department for International Health, University of Tampere, School of Medicine, Tampere, Finland; 4 Department of Paediatrics, Copenhagen University Hospital Rigshospitalet, Copenhagen, Denmark; University of Tokyo, Japan

## Abstract

**Background:**

Malnourished children have increased risk of dying, with most deaths caused by infectious diseases. One mechanism behind this may be impaired immune function. However, this immune deficiency of malnutrition has not previously been systematically reviewed.

**Objectives:**

To review the scientific literature about immune function in children with malnutrition.

**Methods:**

A systematic literature search was done in PubMed, and additional articles identified in reference lists and by correspondence with experts in the field. The inclusion criteria were studies investigating immune parameters in children aged 1–60 months, in relation to malnutrition, defined as wasting, underweight, stunting, or oedematous malnutrition.

**Results:**

The literature search yielded 3402 articles, of which 245 met the inclusion criteria. Most were published between 1970 and 1990, and only 33 after 2003. Malnutrition is associated with impaired gut-barrier function, reduced exocrine secretion of protective substances, and low levels of plasma complement. Lymphatic tissue, particularly the thymus, undergoes atrophy, and delayed-type hypersensitivity responses are reduced. Levels of antibodies produced after vaccination are reduced in severely malnourished children, but intact in moderate malnutrition. Cytokine patterns are skewed towards a Th2-response. Other immune parameters seem intact or elevated: leukocyte and lymphocyte counts are unaffected, and levels of immunoglobulins, particularly immunoglobulin A, are high. The acute phase response appears intact, and sometimes present in the absence of clinical infection. Limitations to the studies include their observational and often cross-sectional design and frequent confounding by infections in the children studied.

**Conclusion:**

The immunological alterations associated with malnutrition in children may contribute to increased mortality. However, the underlying mechanisms are still inadequately understood, as well as why different types of malnutrition are associated with different immunological alterations. Better designed prospective studies are needed, based on current understanding of immunology and with state-of-the-art methods.

## Introduction

**Figure 1:**
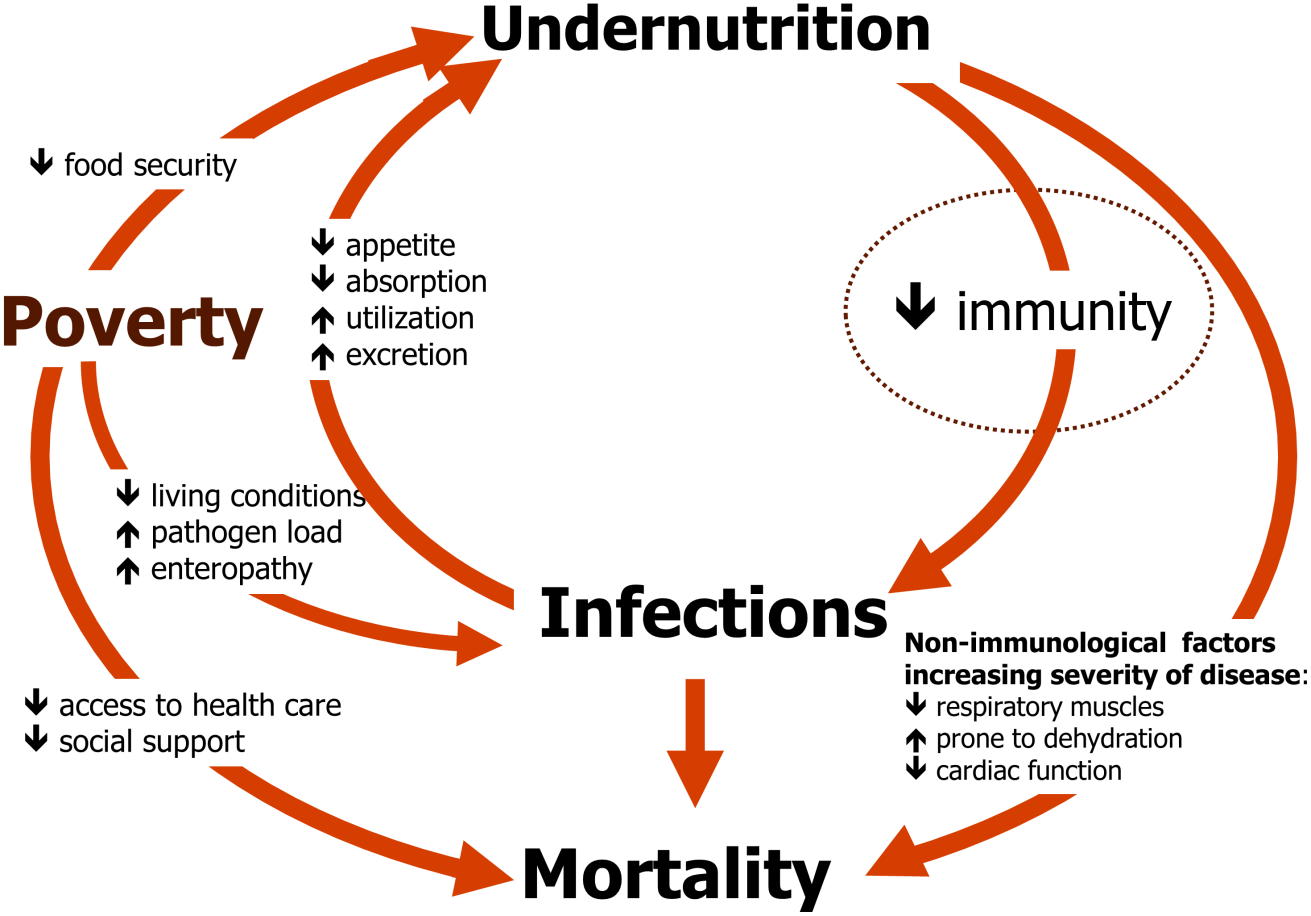
Conceptual framework on the relationship between malnutrition, infections and poverty.

Malnutrition in children is a global public health problem with wide implications. Malnourished children have increased risk of dying from infectious diseases, and it is estimated that malnutrition is the underlying cause of 45% of global deaths in children below 5 years of age [1]–[2]. The association between malnutrition and infections may in part be due to confounding by poverty, a determinant of both, but also possibly due to a two-way causal relationship (**Figure 1**): malnutrition increases susceptibility to infections while infections aggravate malnutrition by decreasing appetite, inducing catabolism, and increasing demand for nutrients [3]. Although it has been debated whether malnutrition increases incidence of infections, or whether it only increases severity of disease [3], solid data indicates that malnourished children are at higher risk of dying once infected [2]–[4]. The increased susceptibility to infections may in part be caused by impairment of immune function by malnutrition [5]. The objective of this study was to investigate the associations of different types of malnutrition with immune parameters in children, through a systematic review of the literature.

Since most infections and deaths in malnourished children occur in low-income settings, the organisms causing disease are rarely identified. Therefore, little is known about whether these differ from pathogens infecting well-nourished children, and whether malnourished children are susceptible to opportunistic infections. Although opportunistic infections like *Pneumocystis jirovecii* and severe varicella has been reported in malnourished children [6]–[7], these studies were carried out before the discovery of HIV, and may represent cases of un-diagnosed paediatric AIDS. More recent studies have found that *Pneumocystis jirovecii* pneumonia is not frequent in malnourished children not infected with HIV [8]. However, quasi-opportunistic pathogens like cryptosporidium and yeast are frequent causes of diarrhoea in malnourished children [9], and malnourished children have a higher risk of invasive bacterial infections, causing bacterial pneumonia [8], bacterial diarrhoea [10]–[11], and bacteraemia [12]–[14], with a predominance of gram negative bacteria. Due to the high prevalence of invasive bacterial infections, current guidelines recommend antibiotic treatment to all children with severe acute malnutrition, even though the evidence behind is not very strong [14].

Non-immunological factors may also contribute to increased mortality in malnourished children: reduced muscle mass may impair respiratory work with lung infections [15]; reduced electrolyte absorption from the gut [16] and impaired renal concentration capacity may increase susceptibility to dehydration from diarrhoea [5]; and diminished cardiac function may increase risk of cardiac failure [17]. Thus, immune function may only be one of several links between malnutrition, infections and increased mortality, but most likely an important one.

### Definitions of malnutrition

This review considers childhood malnutrition in the sense of under-nutrition, causing growth failure or weight loss, or severe acute malnutrition, either oedematous, or non-oedematous.

Growth failure caused by malnutrition has commonly been defined by low weight-for-age (underweight), length-for-age (stunting), or weight-for-length (wasting) [5]. Generally, older studies diagnosed malnutrition using weight-for-age, while newer studies tend to use weight-for-length. Recently, mid-upper arm circumference (MUAC) has been promoted to diagnose severe acute malnutrition, because of its feasibility and because it predicts mortality risk better than other anthropometric indices [18]. Other definitions of malnutrition include specific micronutrient deficiencies, intra-uterine growth restriction, and obesity, but these conditions are outside the scope of this review.

### Severe Acute Malnutrition

**Figure 2:**
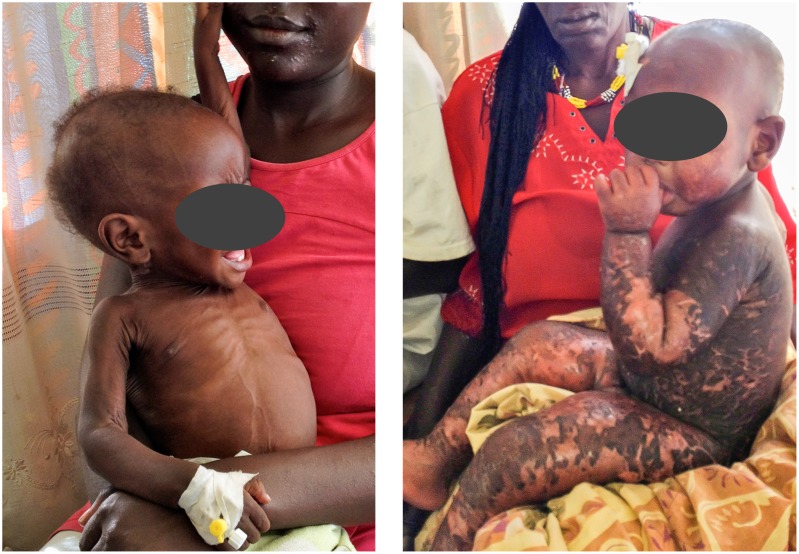
Clinical picture: two forms of severe acute malnutrition, oedematous and non-oedematous malnutrition.

Two forms of severe acute malnutrition in children exist: non-oedematous malnutrition, also known as marasmus, characterized by severe wasting and currently defined by weight-for-length z-score <−3 of the WHO growth standard, or MUAC <11,5 cm; and oedematous malnutrition defined by bilateral pitting oedema (**Figure 2**) [19]. Kwashiorkor refers to a form of oedematous malnutrition, the fulminant syndrome including enlarged fatty liver, mental changes as well as skin and hair changes [20]. The term “marasmic kwashiorkor”, has been used to describe children with both wasting and oedema [21]. It is still unknown why some children develop oedematous malnutrition, and unclear whether this form of malnutrition is associated with a different degree of immune deficiency.

## Materials and Methods

**Figure 3:**
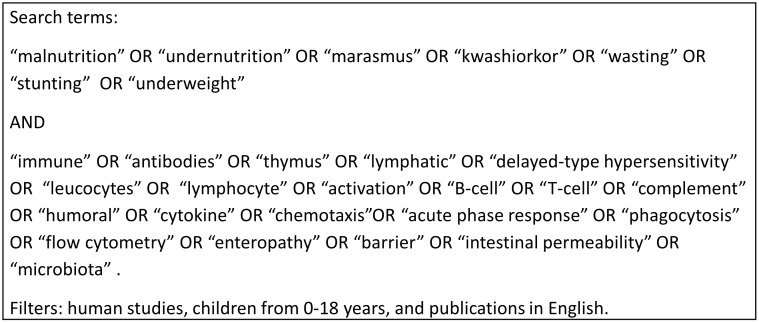
Full search strategy in PubMed, including search terms and filters.

A systematic literature search was carried out in PubMed using combinations of the search terms related to malnutrition and immune parameters. The full search strategy and the search terms used are described in **Figure 3**.

Inclusion criteria were: studies presenting original clinical data regarding immune parameters in children, aged 1–60 months, where a comparison was made, either between malnourished and well-nourished children, or between malnourished children before and after nutritional rehabilitation. Exclusion criteria were studies of children with another primary diagnosis such as cancer, congenital heart disease or endocrine disease. Studies were accepted where children had co-morbid infections, since this is typically seen in malnourished children. Articles by RK Chandra were excluded, due to concerns about possible fraud [22]. Studies published in peer-reviewed scientific journals, as well as in books were included. Only articles in English were included.

The search was carried out in August 2013, and updated in December 2013. The search results were sorted by MJHR, based on titles, abstracts or full-text-articles. Additional literature was obtained from reference lists, text books and by personal communication with experts.

For data retrieval, studies were sorted according to whether they investigated barrier function (skin and gut), innate immunity or acquired immune system, and listed in tables based on the specific immune parameter studied. Some studies were included in more than one table. The following data was extracted from each article: year and country, number and age range of malnourished and well-nourished participants, type of malnutrition and whether included children fulfilled WHOs current diagnostic criteria for severe acute malnutrition, whether infections were present, immune parameter studied, methods used, how the parameter was associated with malnutrition, and whether children with oedematous and non-oedematous malnutrition were differentially affected.

The results of the included articles were summarized for each immune parameter. Due to the heterogeneous nature of study designs, participants and outcomes, it was not meaningful to synthesize the results in a meta-analysis. The main potential bias was presence of infection. For this reason, presence and effect of infection was considered for each study as well as for each outcome. The PRISMA (Preferred Reporting Items for Systematic Reviews and Meta-Analyses) guideline was followed, except for the items relating to meta-analysis (**Checklist S1**).

## Results

**Figure 4:**
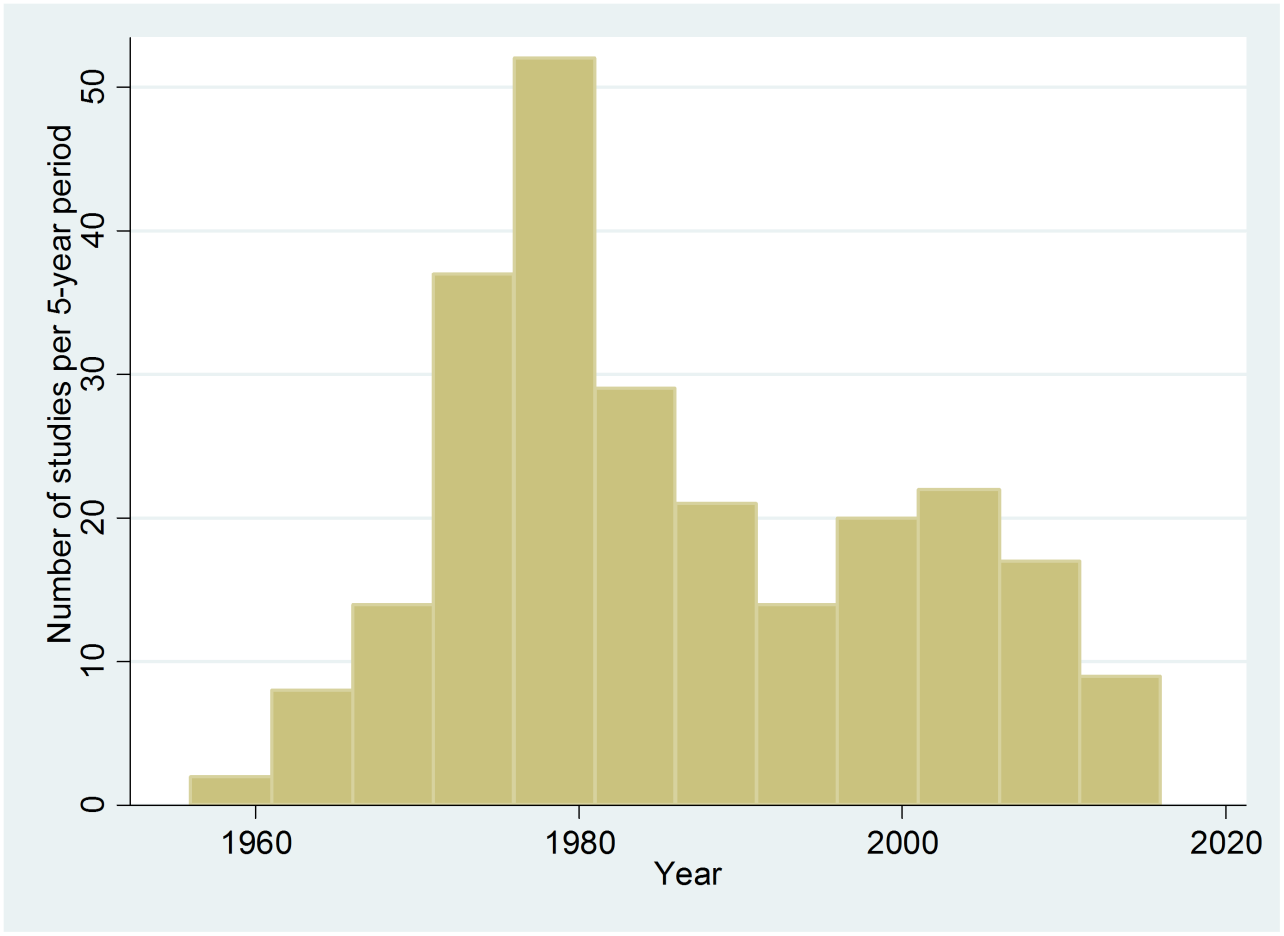
Number of studies published per 5-year period about immune function in malnourished children.

The search in PubMed yielded 3402 articles. By contacting experts in the field, an additional 631 papers were obtained. Reference list of all papers read were screened for relevant papers not included in the initial search. Of all the screened papers, 245 met the inclusion criteria (**Figure S1**). Another 49 articles were identified which, in addition to children 1–60 months old, also included older children. These studies were not included in the main analysis, but used in a sensitivity analysis in which all studies were included. The result of this additional analysis was essentially similar to the results obtained with studies only including children less than 60 month (results not shown). The studies were published between 1957–2014, mainly in the 1970s and 1980s. Only 33 studies were published after 2003 (**Figure 4**). The studies included 29 prospective studies that compared malnourished children to themselves after nutritional recovery, and 216 cross-sectional studies. Of the cross-sectional studies, 51 were community-based, comparing immune parameters in children according to nutritional status. The remaining 165 cross-sectional studies compared hospitalised malnourished children to well-nourished children, often recruited outside the hospital. In 53 studies, all children fulfilled WHOs diagnostic criteria for severe acute malnutrition [23]. The vast majority of these studies included children with oedematous malnutrition, while only two studies included children with non-oedematous malnutrition based on the new WHO growth standard.

The results of each immune parameter are summarized in **Table 1**, and the results of individual articles are summarized in **Tables S1–14**.

**Table 1:** Summary of results in studies of each immune parameter.

Immune parameter	Number of studies	Period	In children with severe malnutrition?	In children with moderate malnutrition?	Different in OM compared to NOM?	Comments	Listed in table
**Skin**	6	1968–1989	- Atrophy - Cells in skin abrasions: ↑ GRAN, ↓ monocytes	Not assessed	Cells in abrasions only affected in OM		S1
**Gut function**	21	1965–2013	Thin mucosa, shorter villi, infiltration of immune cells. Increased intestinal permeability.	No linear relationship	↕	Also in well-nourished children	S2
**Factors in secretions**	19	1968–2012	sIgA in saliva, tears, nasal washings: ↓ in duodenal fluids: ↕, in urine: 0 Lysozyme: ↕. Gastric juice and acidity: ↓	↕	Saliva flow ↓ in OM; sIgA ↑ in OM		S3
**Microbial flora**	11	1972–2014	Different pattern of stool micro-biota; bacterial growth in small intestine; ↑ yeast and g. neg. bact.	↑ yeast in mouth	Not assessed		S4
**White blood cells**	38	1964–2009	Leukocytes in blood: 0; Microbicidal activity ↓, Chemotaxis ↓; Phagocytosis: ↕.	Leukocytes in blood: 0; NK cells: 0	Bactericidal activity ↓ in OM		S5
**Acute phase**	24	1970–2006	Positive APP ↑, negative APP↓ with infection, sometimes also without clinical infection	Few studies	↕		S6
**Complement**	24	1973–2011	C3, C6, C1, C9, Factor B: ↓; C5, C activity: ↕; C4: 0	Not affected	All parameters ↓ in OM	Signs of in-vivo consumption in OM	S7
**Lymphatic tissue**	12	1956–2009	Thymic atrophy. Fewer lymphocytes in thymus cortex. Less atrophy of other lymphatic tissue.	Linear relationship of thymus size with nutritional status	↕	Also ↓ by infections and zinc deficiency	S8 and S9
**DTHR**	21	1965–2092	Mantoux after BCG vaccination: ↓; Reaction to other antigens: ↓	: ↕;	↕	Also ↓ by infections and zinc deficiency	S10
**Lymphocytes**	58	1971–2009	Total lymphocytes 0; T-cells: 0/↑; CD4 count: 0/↑; B-cells: ↓; Response to PHA: ↓	Not affected	CD4 count ↑ in OM	Conflicting result by flow cytometry older methods	S11
**Antibody levels**	32	1962–2008	IgG: 0; IgM: 0; IgA:↑; IgE: ↕; IgD: ↑ in OM, ↕ in NOM	Not affected	IgA and IgD ↑ in OM		S12
**Vaccination response**	35	1957–2009	Antibody titre:↓; Most acceptable sero-conversion; Possibly delay in antibody response	Antibody titre: 0; Sero-conversion: 0	Titres: ↓ in OM		S13
**Cytokines**	35	1975–2013	Th1-cytokines: IL1. Il2, IL12, IFN-γ: ↓; Th2 cytokines: IL10, IL14: ↑; Inflammatory cytokines (IL6, TNFα): ↕	Few studies: Same pattern as severe malnutrition	Th2-response↑, IL6 ↑ in OM; Altered leukotrienes		S14

**Legend**: ↑ =  higher in malnourished than well-nourished, ↓ =  lower in malnourished than well-nourished, 0 =  similar in malnourished and well-nourished; ↕ = inconsistent results; OM =  Oedematous malnutrition, NOM =  Non-oedematous malnutrition, GRAN = Granulocytes (Polymorph nuclear cells); sIgA =  secretory immunoglobulin A; NK =  Natural killer; APP =  Acute phase protein; C = Complement component, BCG =  Bacille Calmette-Guérin, PHA: phyto-hemaglutinin; Ig =  immunoglobulin; IL =  Interleukin; IFNγ = Interferon-gamma; TNFα = Tumour-necrosis-factor-alpha.

### Epithelial barrier function

The barrier function of the skin and mucosal surfaces is considered the first-line defence of the immune system, upheld by the physical integrity of the epithelia, anti-microbial factors in secretions (e.g. lysozyme, secretory IgA and gastric acidity) and the commensal bacterial flora [24].

Of the articles describing barrier function in malnourished children, six described skin structure and function, 21 described structure and permeability of intestinal mucosa, 19 protective factors in secretions and 11 the microbial flora colonizing mucosal surfaces.

#### Skin

*Skin barrier* has mostly been studied in children with oedematous malnutrition, who may develop a characteristic dermatosis, characterized by hyper-pigmentation, cracking and scaling of the epidermis, resembling “peeling paint”, providing a potential entry port for pathogens [25].

Six articles assessed barrier and immune function of the skin in malnourished children (**table S1**). Two articles describing histology reported atrophy of skin layers, but did not describe cutaneous immune cells [26]–[27]. Four articles described the “cutaneous inflammatory response”: They made small abrasions in the skin, and placed microscopy slides over the sites. Similar or higher numbers of white blood cells migrated onto slides in malnourished children, predominantly granulocytes and a lower proportion of monocytes and macrophages [28]–[31]. This pattern was noted to resemble a neonatal immature immune response [30]. All four articles found this pattern in patients with oedematous malnutrition, while one study found that the response of non-oedematous children resembled that of well-nourished [30].

#### Structure and function of the intestinal mucosa

The intestinal mucosa of malnourished children was described in 21 articles (**table S2**). Autopsy-studies from as early as 1965 described a thin-walled intestine in malnourished children, and noted that “… the *tissue paper intestine* of kwashiorkor is well known to tropical pathologists.” [32]. Small-intestinal biopsies showed thinning of the mucosa [33]–[36], decrease in villous height [37]–[43], altered villous morphology [32] [40] [44] and infiltration of lymphocytes [32] [34]–[38]. Electron-microscopy studies found sparse brush border with shortened microvilli and sparse endo-plasmatic reticulum [42]. Others found increased intestinal permeability to lactulose [45]–[48]. Such an intestine may predispose to bacterial translocation, and likewise, one of the included articles described high levels of lipopolysaccharide in the blood of malnourished children, probably originating from gut bacteria translocating into the bloodstream [49]. However, the mucosal atrophy and functional changes did not only occur in malnourished children. Although sometimes found to be most severe in malnourished children [33] [35]–[36] [46]–[47], similar abnormalities were present in apparently well-nourished children from the same environment [38]–[40] [43] [50], and frequently persisted after nutritional recovery [34] [37] [51].

Two articles described immune cells in small intestinal biopsies from malnourished children in Gambia and Zambia: both reported increased lymphocyte infiltration, more T-cells, and cells expressing HLA-DR in malnourished children compared to English children [37]–[38]. However, it was similar to Gambian well-nourished children [38], and unaltered by nutritional recovery [37]. Both well-nourished and malnourished Gambian children had high levels of intestinal cytokine expression, but malnourished children had an increased ratio of cells expression pro-inflammatory to regulatory cytokines, compared to the well-nourished Gambian children [38].

The colon was only described in one article, reporting increased vascularity, atrophy of the mucosa and a tendency to rectal prolapse in children with oedematous malnutrition [52].

Four articles compared the intestine of children with oedematous and non-oedematous malnutrition: one study from South Africa found that the histological changes were most severe in those with oedema [40]. Two articles from Chile found that children with non-oedematous malnutrition had a thinner mucosa, whereas children with oedema had more villous atrophy and more cellular infiltration [35]–[36]. In contrast, a more recent study from Zambia found higher numbers of T-cells and cells expressing HLA-DR in the intestines of children with non-oedematous than oedematous malnutrition, while the intestines of oedematous children were deficient in sulphated glycosaminoglycan [37].

#### Antimicrobial factors in mucosal secretions

Nineteen articles were published on anti-microbial factors in secretions from malnourished children (**table S3**). Secretory IgA (sIgA) was investigated in 15 studies, of which 11 investigated saliva, urine, tears, nasal washings and duodenal fluid [53]–[63] and three investigated small intestinal biopsies [39]–[40] [64].

SIgA in saliva, tears and nasal washings was frequently reduced in severely malnourished children [54]–[55] [57]–[58]. One article from Egypt reported increased levels in children with oedematous malnutrition [56], but may have overestimated sIgA, since saliva flow was reduced in malnourished children, and sIgA was expressed as g/l, whereas other articles expressed it as sIgA as % of protein content. Studies of sIgA in duodenal fluid showed conflicting results [57] [59], as did studies quantifying sIgA in small intestinal biopsies [39]–[40] [64]. The sIgA content of urine was increased or normal in severely malnourished children [60]–[61]. In mild to moderately underweight children, inconsistent results were found for sIgA in tears [63] and saliva [53]–[54] [62]–[63].

Tear lysozyme content was found to be reduced in malnourished children [54] [63], while saliva lysozyme was unaffected [53]–[54]. Gastric acid secretion was consistently reduced in severely malnourished children [65]–[68], and higher pH was associated with bacterial colonization of the stomach [65].

#### Microbial colonization

Microbes colonizing skin and mucosa may protect against infections by competing with pathogens, by producing specific antimicrobial substances, and by stimulating host immune function [69]. Despite much recent interest in the subject, of 11 articles describing the micro-flora in malnourished children, only four were published during the last ten years (**table S4**). All found malnourished children to host a different flora from well-nourished children. Their mouths and throats contained more yeast [70]–[72], and their stomach and duodenum, which in healthy children is considered to be almost sterile, contained a large number of microorganisms [72]–[75]. Although one study found similar degree of small intestinal bacterial overgrowth in diarrhoeal patients with and without malnutrition [75], another found more small intestinal bacteria in malnourished than in well-nourished children with diarrhoea [72]. While gram positive cocci predominated in the small intestine of well-nourished children, malnourished children hosted more gram negative bacteria [65] and yeast [74].

The colonic flora, containing the vast majority of commensal bacteria, was described by sequencing bacterial DNA from stool samples in four recent articles, which consistently found that the pattern of bacteria was different in malnourished and well-nourished children [76]–[79]. More bacteria with pathogenic potential were found in the malnourished children [77]–[78], and their flora was less mature [79] and less diverse [76] [78]. A twin study from Malawi suggested that micro-flora pattern could also play a role in developing malnutrition [76]. No articles have so far reported whether the intestinal flora is different in children with oedematous and non-oedematous malnutrition.

### Innate immune system

The innate immune system delivers an unspecific response relying on leukocytes (like granulocytes, monocytes and macrophages), as well as soluble factors in blood (like acute phase proteins and the complement system) [24]. Of the articles describing innate immune response, 38 described number and function of leucocytes, 25 acute phase proteins and 24 complement components and activity.

#### White blood cells of the innate immune system

Thirty-eight articles described number and function of leukocytes of the innate immune system (**table S5**). Most reported similar or higher numbers of total leukocytes in blood of malnourished children [49] [80]–[92], and three found that granulocytes were higher in malnourished children [81] [86] [93].

Two studies from Nigeria and one from Ghana found no difference in the mean percentage of natural-killer-cells among malnourished or well-nourished children [94]–[96], although two reported that more malnourished children had abnormally low numbers of natural-killer cells. In Zambia, levels of dendritic cells were lower in blood from malnourished children before nutritional rehabilitation than after, and elevated inflammation markers were associated with a paradoxical lower level of dendritic cell activation. This was associated with endotoxin levels in the blood, and was interpreted as a type of immune-paralysis, related to inflammation and bacterial translocation [49]. Unfortunately, it was not assessed whether this was different from well-nourished children with severe infections.

Chemotaxis of granulocytes was reduced in malnourished children in three of five studies [80] [83] [97]–[99], and one study found a diminished ability to adhere to foreign material [100]. Results for phagocytosis were mixed: five of 12 studies found that leukocytes of malnourished children had reduced ability to ingest particles or bacteria [81] [83] [88]–[89] [97]–[98] [101]–[106]. Microbicidal activity of granulocytes was reduced in malnourished children in five of seven studies [80] [83] [88] [97]–[98] [103] [107], while two of three studies found macrophages from malnourished children to have normal microbicidal activity [89] [108]–[109]. Neutrophils may kill microorganisms by producing reactive oxygen compounds; assessable by the Nitroblue Tetrazolenium (NBT) test, which, however, gave inconsistent results in malnourished children [83] [105] [110]–[114]. It has been hypothesized, that reactive oxygen production is involved in the pathogenesis of oedematous malnutrition [115]; however, the NBT test results did not show any clear pattern in children with oedematous compared to non-oedematous malnutrition.

One study found the levels of enzymes, like alkaline and acid phosphatase, to be increased in leukocytes from children with malnutrition [116]. More leukocytes of malnourished children were found to have markers of apoptosis (CD95) [92], and signs of DNA damage [117]–[118].

No articles have yet described the expression of pattern-recognition molecules, like Toll-like receptors in malnourished children, although these are fundamental to the function of the innate immune system.

#### Acute phase response

Acute phase responses is induced by infection or trauma, and mediated by cytokines like IL-6 and TNF-α. It involve temporal suppression of acquired, and amplification of innate immune responses, with secretion of positive acute phase proteins (APP) like C-reactive protein (CRP), serum-amyloid-A (SAA), complement factors, α-1-acid-glycoprotein or ferritin [119], while levels of other proteins are reduced, as albumin, pre-albumin, transferrin, α -2-HS-glycoprotein, and α -fetoprotein. These are sometimes called ‘negative acute phase proteins’, although it is not clear whether their reduced level are due to active down-regulation, or because of competition with production of positive acute phase proteins. Twenty-four articles described the levels of acute phase proteins in malnourished children with or without infection (**table S6**).

#### Acute phase response in children with infections

Most studies found elevated positive APP in malnourished children with infections. This included CRP [120]–[128], α-1 acid-glycoprotein [120]–[121] [129], haptoglobin [120]–[121] [125] [127] [129] while the results for ceruloplasmin [125] [130], and α-1-antitrysin were inconsistent [120]–[121] [125] [127]–[129]. Only one study found lower CRP levels in malnourished than well-nourished children with similar infections, despite higher levels of IL-6 [129]. So-called negative APP were uniformly low in children with malnutrition and infection, including transferrin [94] [127] [130]–[133], α-2-HS-glycoprotein [134]–[136], pre-albumin [122], fibronectin [132], and α-2-macroglobulin[127].

#### Acute phase response in children without infections

Three studies found elevated CRP in malnourished children without apparent infections [94] [124] [128], while two studies found similar CRP-levels in malnourished and well-nourished children [122] [137]. Results for α-1-antitrysin were inconsistent [128]. So-called negative acute phase proteins like transferrin [94] [130], α-2-HS-glycoprotein [135], fibronectin [133] [138] and pre-albumin [122] [138]–[139] were consistently reduced in malnourished children, even without infections.

#### Acute phase response to a controlled stressor

Four articles described the acute phase response induced by a vaccine. Two reported a normal [140] or increased [141] febrile response to measles vaccine in malnourished children. In another study, a similar rise in APP was seen in malnourished and well-nourished children [137], in response to a diphtheria-pertussis-tetanus-vaccination, but the increase in APP was greater when the vaccination was repeated after nutritional rehabilitation. The same was found for the febrile response to a repeated vaccine in malnourished children [142]. Since no repeated vaccine was given to well-nourished children, it is unknown whether they would also have had a stronger response to the second dose.

#### Complement

The complement system consists of plasma proteins secreted by the liver that, upon activation, react to recruit immune cells, opsonize and kill pathogens [24]. Three main pathways activate the complement system: the classical pathway, the alternative pathway and the lectin pathway [143], with the complement protein C3 playing a central role in all three pathways.

Twenty-four articles described levels or in-vitro activity of complement proteins (**table S7**). In 17 of 21 studies, levels of C3 were depressed in malnourished children [89] [94] [99] [106] [124]–[125] [127]–[130] [144]–[154]. Two studies found C3 to correlate with albumin [94] [148], and with one exception [94], C3 levels were lower in children with oedematous than non-oedematous malnutrition [89] [146] [149] [150]–[151] [153].

Few studies assessed C6, C9, and factor B, and most found reduced levels in malnourished children [145] [148]–[149] [151] [153], most so in oedematous malnutrition [148]–[149] [151] [153].

Levels of C1 and C4 were mostly normal in malnourished children [94] [99] [145] [148] [150]–[153], while two studies found reduced levels of C4 in patients with oedematous, but not non-oedematous malnutrition [89] [149]. Studies assessing C5 showed inconsistent results [145] [148]–[149] [151] [153].

Classical pathway activity was either unaffected [106] [145]–[146] [152], reduced [148] [155] [156], or reduced only in oedematous, but not in non-oedematous malnutrition [157]. Alternative pathway activity was reduced in two studies [145] [156] and unaffected in one [146]. General opsonic activity of serum was reduced in one study [156]. No articles reported the activity of the lectin pathway.

Both reduced production and increased consumption may explain the reduced levels of complement factors. Complement components are produced by the liver, and their levels correlated with albumin levels, the production of which is also impaired in malnutrition [158]. However, increased consumption is also supported by one study showing high levels of C3d, a by-product after activation of C3, in malnourished children, most pronounced in oedematous malnutrition [148].

### Acquired immunity

Acquired immunity is characterized by specialized cellular and antibody-mediated immune responses, generated by T- and B-lymphocytes reacting with high specificity towards pathogens and creating long-lasting immunological memory. The acquired immune system also orchestrates tolerance to self and other non-pathogenic material like gut bacteria [24]. Of the articles describing acquired immunity, 12 described the thymo-lymphatic system, 21 delayed-type hypersensitivity responses (DTHR), 58 lymphocyte subsets in blood, 32 immunoglobulins in blood, 35 vaccination responses and 35 cytokines.

#### Thymus

The thymus gland is the central lymphatic organ in the acquired immune system, where maturation and proliferation of T-lymphocytes take place. The thymus is large at birth and undergoes gradual involution after childhood [159], with diminished output of T-lymphocytes [160].

Six articles reported autopsy studies of the thymo-lymphatic system in malnourished children, published between 1956 and 1988 [161]–[166] **table S8**). All reported thymus atrophy in malnourished children, to an extent termed “nutritional thymectomy” [164]. Histology revealed depleted thymocytes, replacement with connective tissue, and decreased cortico-medullar differentiation [163] [165]–[166].

Eight articles reported thymic size measured by ultrasound, in relation to nutritional status [91] [167]–[173] (**table S9**). Five of these studied children with severe malnutrition and found severe thymic atrophy [91] [167]–[170], reversible with nutritional rehabilitation, although thymic size did not reach normal levels as fast as anthropometric recovery [91] [170]. Thymic size was also measured by ultrasound in cohorts of children to determine patterns of thymic growth [159] [171], in a vaccination trial in Guinea Bissau [172] and in a pre-natal nutritional supplementation trial in Bangladesh [171]. These studies confirmed that thymus size was associated with nutritional status, even in mild malnutrition. Breastfed children often had a larger thymus than artificially fed children [174], possibly explained by IL-7 in breast milk [175], and children with a large thymus were found to have a higher chance of surviving than those with a small thymus [172] [176].

#### Other lymphatic tissue

Six articles reported investigations of other lymphatic tissue. Four autopsy studies found atrophy of lymph nodes, spleen, tonsils, appendix and Peyer's patches, although not as pronounced as in the thymus. Histology revealed a reduction in germinal centres and depletion of lymphocytes from para-cortical areas [161] [163]–[165]. Two studies in living children also found that the tonsils were smaller in malnourished than in well-nourished children [163] [177].

#### Delayed type hypersensitivity response (DTHR)

Cellular immune function can be examined by dermal DTHR, the prototype of which is the Mantoux test. Intradermal application of substances like candida or phyto-hemaglutinin (PHA) are also used, as well as sensitizing skin with a local contact sensitizer such as 2-4-di-nitro-clorobenzene (DNCB). Twenty-one articles reported DTHR in relation to malnutrition (**table S10**).

The majority of studies found that malnourished children less frequently developed a positive Mantoux after BCG vaccination [154] [177]–[185]. Most also found diminished reactivity to *Candida*, PHA and other common antigens [29] [145] [179] [183] [186]–[190], and after sensitizing with DNCB [163] [177] [179] [183] [188] [191]–[192]. Conflicting results were found for DTHR in children with different types of severe malnutrition: Three studies found most impaired response in oedematous malnutrition [179] [181] [191], while one found that it was worst in non-oedematous malnutrition [184], and two studies found similar responses [186] [187].

The proportion of positive DTHR varied from study to study, both in well-nourished and malnourished children. Inconsistent results were found in moderately malnourished children [178] [180]–[181] [185]–[187] [193]. Other studies found that DTHR was improved with zinc supplementation [190] [194]–[195] diminished by infections [178] [181] [196], and in slightly older children, a strong interaction was seen between infections and nutritional status [197].

#### Lymphocytes in blood

Fifty-eight articles reported either total numbers of lymphocytes or lymphocyte subsets in blood (**table S11**). Of 16 articles, 13 reported similar or higher levels of lymphocytes in peripheral blood of malnourished children [80]–[83] [85]–[87] [90] [93] [101] [177] [179] [187] [191] [198] [199].

Three studies found that children with oedematous malnutrition had more atypical lymphocytes in blood, resembling plasma cells [81] [87] [93]. Other indicators of functional differences were higher density [200], different pattern of gene expression [201], and more markers of apoptosis in lymphocytes of malnourished children [92] [202].

#### T-lymphocytes in blood

Numbers of T-lymphocytes were described in 29 articles (**table S11**). Early studies identified T-lymphocytes as those forming rosettes with sheep red blood cells, while later studies used monoclonal antibodies to CD3. Using the rosette-method, 19 of 20 studies found lower levels of T-lymphocytes [28] [87] [93] [101] [128] [130] [144] [183] [186]–[187] [191] [199] [203]–[210]. Four studies using monoclonal CD3-antibodies and cell-counting by microscopy also found reduced levels of T-lymphocytes in malnourished children [144] [167]–[168] [207]. In contrast, only one flow cytometry study found lower levels of T-lymphocytes in malnourished children [211], while four did not [86] [94] [210] [212]. Accordingly, it seems like the rosette-based method identifies different T-lymphocytes than flow cytometry. Some studies found that the numbers of T-lymphocytes were reduced in acute infections [86] [90] [212].

#### Lymphocyte response to PHA stimulation

In healthy children, incubation of lymphocytes with PHA results in T-lymphocytes to proliferate. Seventeen out of 23 articles reported a reduced proliferative response to PHA in lymphocytes of malnourished children [93] [97]–[98] [101] [147] [154] [163] [177] [179] [186]–[187] [189]–[190] [192] [196] [203] [212]–[218]. Zinc supplementation improved the response in malnourished children [190].

#### CD4+ lymphocytes

With assessment of CD4 counts becoming widely available, it has been investigated whether the number of CD4+ lymphocytes was affected by malnutrition. In children without HIV, two of four studies using monoclonal antibodies and microscopy found reduced levels of CD4+ lymphocyte in malnourished children [144] [168] [219] [207], while all seven flow cytometry-studies except one [211] found similar or higher levels [86] [90] [91] [94] [198] [212]. Bacterial infections were noted to reduce the CD4-count [86]. For malnourished children infected with HIV, it was hoped that re-nutrition alone could increase their level of CD4+ lymphocytes. However, a study from Zambia found that CD4 counts declined during nutritional rehabilitation in HIV-infected malnourished children without anti-retroviral treatment [198]. Thus, a low level of CD4+ lymphocytes can probably not be attributed to malnutrition, regardless of whether the child has HIV or not.

Three studies noted that level of CD4+ lymphocytes were higher in children with oedematous than with non-oedematous malnutrition [91] [198] [220], and several studies have noted that children with HIV were less likely to develop oedematous malnutrition [198] [220] [221], suggesting that some level of CD4+ lymphocytes could be required to develop the syndrome.

#### Activation markers on T-lymphocytes

Most flow cytometry studies assessing surface markers on T-lymphocytes have been carried out in Mexico, all comparing malnourished infected children with similarly infected well-nourished children. Malnourished children were found to have fewer effector T-lymphocytes, identified as cells lacking the “naïve” markers CD62L and CD28 [90], fewer activated T-lymphocytes, with the markers CD69 and/or CD25 [212] [222] [223], and fewer memory T-lymphocytes identified by the marker CD45RO+ [86]. In contrast, a study from Ghana found similar numbers of activated T-lymphocytes, identified by HLA-DR, in malnourished and well-nourished children [94].

#### B-lymphocytes

Articles published before 1990 measured B-lymphocytes as those forming rosettes when incubated with sheep erythrocytes and C3, while more recent studies used monoclonal antibodies to CD20 and flow cytometry. All seven rosette-based studies found unaffected or higher B-lymphocyte counts in malnourished children [130] [186] [200] [204] [206] [213] [224], as did one study using anti-CD20 and microscopy [167]. In contrast, all four studies using flow cytometry found reduced numbers of B-lymphocytes in malnourished children [86] [94] [211] [212].

#### Antibody levels

Thirty-two articles described immunoglobulins in blood of malnourished children (**table S12**). Nineteen of 27 studies found no difference in IgG antibodies or total γ-globulin between malnourished and well-nourished children [94] [53] [63]–[64] [82] [130] [144] [147] [150] [154] [179] [186] [224]–[238]. Likewise, IgM levels were most frequently similar, or higher in malnourished than well-nourished children [94] [53] [63]–[64] [82] [130] [144] [147] [154] [179] [186] [224]–[225] [227]–[238].

IgA was elevated in malnourished children in 19 of 27 studies [94] [53] [55]–[56] [63]–[64] [82] [130] [144] [147] [150] [154] [179] [224]–[225] [227]–[238]. With a few exceptions [150] [232], all studies found elevated levels of IgA in oedematous malnutrition, while 11 of 19 studies found that IgA in non-oedematous or underweight children was normal [94] [53] [55]–[56] [63] [82] [130] [144] [150] [154] [179] [224] [227] [230] [233]–[238]. One study noted that levels of IgA correlated with the degree of dermatosis in children with Kwashiorkor [231].

IgE showed no clear pattern, but was elevated in malnourished children in three of six studies [82] [147] [211] [233] [238]–[239]. IgD, present in low amounts in healthy children, was elevated in children with malnutrition in two studies[130] [233], or elevated in oedematous but not non-oedematous malnutrition [179], while one study found that it was similar to well-nourished children [82].

#### Antibody vaccination responses

Thirty-five articles described vaccination responses to a specific antigen (**table S13**). The articles either reported *sero-conversion rates*, or *antibody titre* response. Studies assessing sero-conversion rates in children with severe malnutrition found mixed results: Six of 10 studies found reduced sero-conversion rates in children with severe malnutrition to typhoid [101] [240], diphtheria [101], tetanus[101] [206], tetanus-diphtheria-pertussis (DTP) [234], hepatitis B [241], measles [141] [149] [242] and yellow fever [243]–[244], and two studies found that sero-conversion was delayed in malnourished children [245] [238]. Ten of 11 studies found that severely malnourished children responded with reduced antibody titres [101] [141] [149] [206] [233]–[234] [238] [240]–[242] [246], despite some of the studies finding acceptable sero-conversion rates. No study found that children with oedematous malnutrition had a normal antibody response to vaccination. One study from 1964 found improved antibody response to DTP in children with oedematous malnutrition randomized to a high-protein diet [247]. There did not seem to be any specific vaccines whose antibody response was more affected than others by malnutrition, nor was there any pattern in terms of responses to live or dead vaccines.

In contrast, mild and moderately malnourished children were most often found to seroconvert normally when vaccinated against smallpox [248], diphtheria [101] [178] [249] [284] , DTP [178] [234], measles [139] [140] [178] [245] [250]–[255], polio [178] [256], meningococcus[178] [257], and hepatitis B [258], and 9 of 11 articles reported similar level of antibody titres response in moderately malnourished, as well-nourished children [101] [140] [154] [178] [234] [248]–[249] [252]–[253] [258]–[259].

Three of five articles reported similar adverse reactions to vaccination in malnourished as in well-nourished [140]–[141] [242] [245]–[255]. In contrast, one study found that malnourished children given measles vaccine frequently developed diarrhoea, pneumonia and fever, compared to well-nourished children, who, in turn, more often developed a rash [141].

Results were inconsistent for studies assessing levels of specific antibodies to non-vaccine antigens, like blood type antigens [260] malaria [261], H. *influenza*, E. *Coli* [235] [262], *Ascaris* [211], Rotavirus and Lipopolysaccharide [262]. Most of these studies were done in children with moderate malnutrition.

#### Cytokines

Cytokines are signal molecules acting locally between immune cells, and sometimes with systemic effects. Thirty-five articles described cytokines in malnourished children (**table S14**).

Early works identified cytokines as factors in serum influencing various in-vitro functions of immune cells. Thus, three of five studies found that “Leucocyte Migration Inhibiting factor” was lower in malnourished children [84] [263]–[264], that serum from malnourished children contained an “E-rosette inhibiting substance” [128] [265], “lympho-cytotoxin” [266], and a substance inhibiting lymphocyte response to PHA [218] [147] [267]–[268], sometimes called IL-1 [269]. Similarly, Interferon (IFN) was quantified by the antiviral effect of plasma on a cell culture [95] [196]. In neither of these bioassays, the substance responsible for the effect was known. More recent studies assessed levels of cytokines by immunoassays, looking for structurally known cytokines in plasma [123] [270]–[272] or in cultured leucocytes [89], with flow cytometry staining for intracellular cytokines [222]–[223], or by identifying mRNA coding for the protein [273]–[274], with remarkably consistent results.

Cytokines commonly found to be low in malnourished children included IL- 1 and IL-2 [222]–[223] [269]–[270] [273]–[274], although one study found both cytokines to be normal in non-oedematous malnutrition and lower in oedematous malnutrition [89]. IFN-γ was low in malnourished children in six studies [49] [222]–[223] [273]–[275], unaltered in malnourished children in one [276], and elevated in one [272]. IL-12 [49] [274], IL-18 and IL-21 [274], and Granulocyte Macrophage Colony Stimulating Factor [270] were also found to be lower in malnourished children. Blunted cytokine response after in-vitro stimulation with LPS was found in malnourished children [276]–[278], while incubation with leptin normalized their pattern of intracellular cytokines [223].

Other cytokines were mostly found to be elevated in malnutrition: IL10 was elevated in four of five studies [49] [222]–[223] [272]–[273], so was IL-4 [211] [273] [276] and soluble receptors to Tumour Necrosis Factor-α [123]. IL-8 was elevated [277] or unaltered [272].

Tumour Necrosis Factor-α (TNFα) [49] [129] [271]–[273] [276] [279]–[280] and IL-6 [120] [122]–[123] [129] [271]–[273] [276]–[278] were mostly similar or higher compared to well-nourished, most often in studies of infected children.

Comparing cytokine pattern between children with oedematous and non-oedematous malnutrition, most found that the difference from well-nourished was greatest in children with oedematous malnutrition [84] [89] [123] [265] [269]–[270] [277], while two studies found no difference between oedematous and non-oedematous malnutrition [218] [271].

Leukotrienes (LT) are not strictly cytokines, but immune modulating molecules derived from long chain polyunsaturated fatty acids. Levels of LTC4 and LTE4 were higher, and LTB4 lower, in children with oedematous than with non-oedematous malnutrition, whose levels were similar to well-nourished [281], and prostaglandin E2 [282] was higher in children with oedematous malnutrition than in well-nourished.

## Discussion

We identified and reviewed 245 articles about immune function in malnourished children. Some general problems apply to many of the studies, mostly related to their observational design. For this reason they can only describe associations, not causalities.

First, many studies were done in severely malnourished children from hospital settings, who were ill with infections, making it difficult to disentangle the immunological effect of malnutrition from the effect of infection. This problem has caused some to propose that there really is no immune impairment by malnutrition, and that all alterations seen are due to infections or underlying unknown immune deficiencies, which are also responsible for the poor growth [283]. Enteropathy could be an example of such an “invisible” condition, causing both immune deficiency and malnutrition. This hypothesis is difficult to test. However, some studies did try to account for this problem by selecting malnourished children without clinical infections, or by comparing them to well-nourished infected children. In studies from central Africa in the 1970s and 1980s, some malnourished children may have suffered from unrecognized paediatric HIV [284], giving obvious problems for interpretation.

Second, publication bias is a well-known problem, and may have occurred, particularly in older studies, where some small studies showed a dramatic effect.

Third, studies used different diagnostic criteria for malnutrition, making it difficult to determine the children's degree of malnutrition as defined by present-day criteria. While children in 52 of the studies fulfilled WHOs present criteria for severe acute malnutrition, only two diagnosed children based on the new WHO growth reference. Those defined as severely malnourished based on old growth references would most likely also be classified as severely malnourished today, since the new WHO standard tend to classify more children as severely malnourished, while some children then defined as moderately malnourished would be classified as severely malnourished today. The studies including children based on weight-for-age probably included children with stunting and wasting, without differentiating between the two.

Fourth, even using uniform criteria, malnourished children are a heterogeneous group. Anthropometric measurements are only crude markers of body composition, which - among other things - reflect nutrient deficiencies. It is unknown what specific nutrients were deficient, and to what extent infection contributed. Deficits in lean tissue and fat tissue are plausibly different physiologic conditions, and children appearing similarly malnourished may be so for entirely different reasons, with different immunological consequences. No articles have so far reported reliable measures of body composition, simultaneously with markers of immune function. Probably, the consequence of malnutrition on immune function may also depend on the pattern and load of infections. Although most studies were carried out in low-income settings with high infectious loads, a few were from middle- or high-income countries. This may also contribute to inconsistencies in the results.

**Figure 5:**
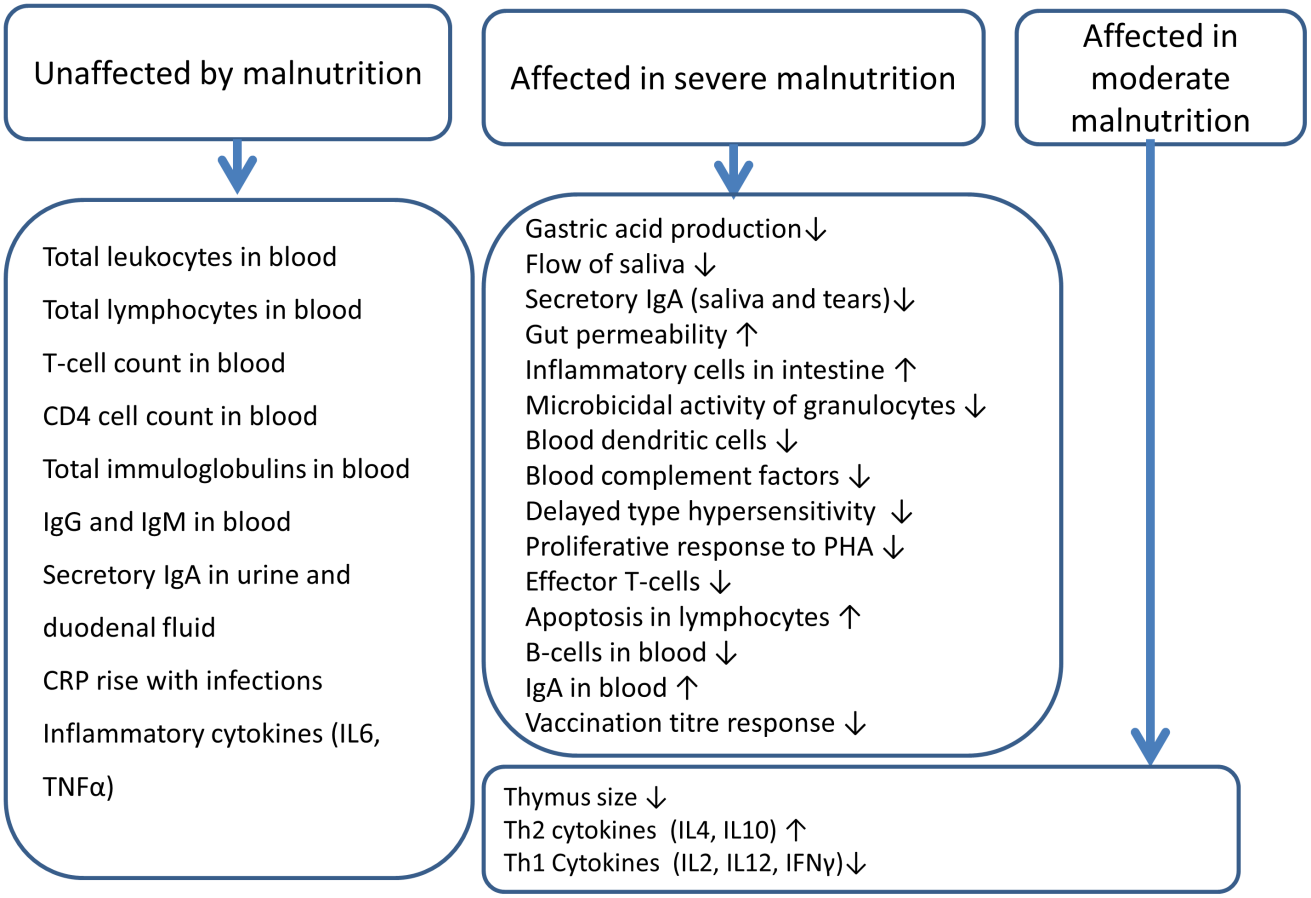
Summary of immune parameters affected and not affected by malnutrition.

In spite of these limitations, common patterns emerge from the studies, summarized below (**Figure 5**).

### Immune parameters apparently not affected by malnutrition

Total white blood cell and lymphocyte counts in peripheral blood are not decreased in malnourished children, and granulocytes are frequently elevated. Likewise, T-lymphocytes and CD4 counts appear normal in malnourished children, when measured by flow cytometry, the gold standard for characterizing cell subsets. Their levels seem to be determined more by infections than by nutritional state, and do not reflect the degree of malnutrition-related immune deficiency, as high infectious mortality is seen in malnourished children, despite unaffected white blood cell counts [49].

Malnourished children can mount an acute phase response to infections, with elevated CRP and low negative acute phase reactants, and this can also be seen in absence of clinical infection. Thus, based on available evidence, the acute phase response, if anything, seems exaggerated rather than diminished. Levels of IgM and IgG are normal or elevated in malnourished children. Secretory IgA is not consistently lower in duodenal fluid, and frequently elevated in urine.

### Immune parameters affected by malnutrition

The gut mucosa is atrophied and permeable in malnourished children. This enteropathy also affects well-nourished children in poor communities, but probably most severely in malnourished children. The condition appears similar to *tropical sprue* described in adults, and the term *enteropathy of malnutrition* has been replaced by the broader term *environmental enteropathy* [285]. At present, this condition is thought to result from high pathogen load rather than nutrient deficiencies, and thus primarily a cause of malnutrition, particularly of stunting [286] [287].

Production of gastric acid and flow of saliva is reduced in malnourished children. Secretory IgA is also reduced in saliva, tears and nasal washings from children with severe, but not moderate malnutrition. The small bowel of malnourished children is often colonized with abundant bacteria, and their pattern of commensal flora is altered. Granulocytes kill ingested microorganisms less effectively. Levels of complement proteins are low in blood from malnourished children, particularly in children with oedematous malnutrition, and less in children with moderate malnutrition.

Lymphatic tissue, particularly the thymus, undergoes atrophy in malnutrition in a dose-response fashion: thymic size depends on nutritional status even in milder degrees of malnutrition, and thymus size is a predictor of survival in children.

DTHR is diminished in malnourished children. Lymphocytes of malnourished children are less responsive to stimulation with PHA, fewer are activated and more cells have markers of apoptosis. Plasma IgA is mostly elevated in malnourished children, particular in those with oedema. Children with severe, but not moderate, malnutrition mount a lower specific antibody response to vaccination, although for most children sufficient to obtain protection. The lower titres seen in malnourished may be due to a delay in vaccination response.

Cytokines can be classified as those promoting a Th1 response of predominantly cellular immunity, and those promoting a Th2-response of humoral immunity [24]. Although this approach has somewhat been replaced by other classifications [288], it seems useful to describe the profile of malnourished children, whose immune system seems tuned towards a Th2 response, with high IL4 and IL10, and low levels of IL-2, IL-12 and IFN-γ. Elevated levels of IL-6 and TNFα may primarily be related to infections, and support the observation that induction of an acute phase response is intact in malnutrition. A more recent classification focuses on whether cytokines are predominantly inflammatory or anti-inflammatory [289]. Malnourished children appear to have high levels of anti-inflammatory cytokines and less clearly affected levels of pro-inflammatory cytokines in blood, in contrast to the predominantly pro-inflammatory cytokine expression in the gut of malnourished children.

### Mechanisms

The mechanisms behind these immunological alterations are still not adequately understood. Some explain it by lack of energy and building blocks to synthesize the proteins required [290]. However, lack of building blocks does not explain why some immune parameters seem intact, or paradoxically elevated in malnutrition, such as plasma IgA, acute-phase proteins, leucocytes in blood, and production of Th2 cytokines. If it was simply a matter of lack of building blocks, all parameters of the immune system should be equally affected. The fact that the pattern of cytokines in malnourished children is tuned towards at Th2- response fits with their high levels of immunoglobulins, reduction in thymus size and diminished DTHR. Still, the pathophysiology behind this Th2 skewedness remains unexplained.

Infections could obviously contribute to the changes seen, and interactions have been noted between infection and malnutrition in their respective effects on immune parameters [197]. However, although many of the immunological changes appear to be synergistically affected by malnutrition and infections, malnutrition also seems to be independently associated with altered immune function.

Animal studies suggest hormonal factors to be involved in the immune profile of malnutrition. Leptin [291], prolactin [292] and growth hormone [293] all stimulate thymic growth and function, and their levels are low in malnourished children. In support of this, a recent study found that a low leptin level was associated with a higher risk of death in malnourished children [272]. Growth hormone therapy increased thymic size and output in adult HIV patients [294]. In contrast, cortisol and adrenalin induce thymic atrophy in mice [295]–[296], and cortisol is high in children with malnutrition and other forms of stress. It is plausible that this hormonal interplay is implicated in the immune deficiency in malnourished children.

This hormonal profile is similar to that of an acute phase response, where thymus atrophy also occurs, acquired immunity is temporarily suppressed and innate immunity takes over [296]. This could explain why some malnourished children have elevated positive APP and most have depressed negative APP in absence of clinical infections. Zinc deficiency causes thymic atrophy [297]–[298], and acute phase responses lower plasma zinc, so zinc status may contribute to the immune deficiency of both malnutrition and acute phase responses.

In HIV infection, persisting subclinical inflammation and immune activation is frequently present, and may be partly responsible for immune deficiency and disease manifestations [299]. Given the frequent finding of elevated acute phase proteins in malnourished children, it seems plausible that a similar state of subclinical inflammation could be involved in both the impairment of immune function, and in the vicious circle of catabolism and deterioration of the nutritional status. However, in spite of elevated acute phase proteins, most studies have reported unaffected or even paradoxically lowered levels of activated T-cell and dendritic cells in malnourished children.

The intracellular receptor, *mammalian target of Raptomycin* (mTOR), is present in most cells. It responds to concentrations of nutrients in the cell's surroundings, and to other signs of stress, such as hypoxia, enabling the cell to adapt its metabolism to locally available nutrients. Immune cells also use mTOR to regulate their state of activation. Nutrient availability may thereby determine whether an immune cell is activated [300], and whether T-cells differentiate towards a pro-inflammatory or a tolerance-inducing phenotype [301]. Some immune cells may even deplete the micro-environment of certain nutrients, to manipulate the activation of mTOR. Accordingly, the significance of nutrients in the micro-environment expands from simple building blocks to signal molecules. Obviously, this mechanism could be involved in the immunological profile in malnutrition. However, no articles have yet described the activity of mTOR in malnourished children.

A research group working with animal models of malnutrition has proposed a theory called the “tolerance hypothesis” [302]. This suggests that the depression of cellular immunity in malnutrition is an adaptive response to prevent autoimmune reactions, which would otherwise occur as a result of catabolism and release of self-antigens. Although adaptive in this sense, it happens at the price of increased susceptibility to infections [303]. However, if this tolerance hypothesis holds true, one would expect to see occasional break-through of auto-immune reactions in malnourished children. Such phenomena have apparently not been studied.

The pathogenesis of oedematous malnutrition is still unknown. Many immune parameters seem affected to a different degree in children with oedematous malnutrition, with higher levels of IgA, higher levels of abnormal antibodies like IgD, poorer vaccination responses and cytokines more skewed towards a Th2-response; their complement levels are lower, which may partly be caused by increased consumption of complement in-vivo. The pattern of leukotrienes is different in children with oedematous compared to non-oedematous malnutrition. This immunological profile resembles that seen in autoimmune diseases such as lupus erythematosus [304]–[305]. Moreover, elevated immunoglobulins in children with oedematous malnutrition seem to correlate with its unexplainable manifestations, like dermatosis and oedema [231] [233]. It could be speculated whether this syndrome could indeed represent some kind of autoimmune reaction to malnutrition, perhaps resulting from a failure to induce efficient tolerance.

## Conclusion

In spite of the prevalence of malnutrition, and its fatal consequences, scientific interest in the immune deficiency of malnutrition seems dwindling, and little research has been carried out on the topic during the last ten years. For this reason, most evidence on the subject relies on immunological methods used 30 to 40 years ago, many of which are no longer in use, and little research has been done with modern methods, and with the present understanding of immunology. Moreover, most studies have looked at isolated aspects of immune function, despite the fact that the parameters are interdependent, and the division into innate and adaptive immune function seems to be a simplification. Thus, our understanding of immune function in malnutrition is still very limited.

This review illuminates the little that we know about the immunological alterations associated with malnutrition, and also points to significant gaps in our knowledge. Future well designed prospective cohort studies should examine how immune parameters are related to morbidity and mortality in malnourished children, with detailed characteristic of nutritional status, preferably body composition, of infections, enteropathy and of low-grade inflammation. When testing nutritional and medical interventions for malnutrition, immune parameters should be included as outcomes. Studies should investigate newer immunological parameters in malnutrition, like expression of innate pattern recognition receptors (as the Toll-like receptor), the lectin pathway of the complement system and mTOR expression and activity. It should be investigated whether a small thymus is associated with lower output of recent thymic-derived T-cells, and how it correlates with hormones like leptin, cortisol, insulin and Insulin Growth Factor-1. Innate and adaptive immune parameters should be assessed simultaneously, taking into account their dynamic interdependency. To understand whether malnutrition is indeed associated with active down-regulation of immune reactivity (as formulated in the “tolerance hypothesis”), the balance between regulatory T-lymphocytes and their counterparts, Th17 lymphocytes should be measured. Finally, prospective studies among children at risk should assess whether immune profiles differ in those who subsequently develop oedematous and non-oedematous malnutrition, and it should be investigated whether children with oedematous malnutrition have markers suggestive of auto-immune or inflammatory diseases. Such studies would reduce our current ignorance on the interplay between malnutrition and infectious diseases.

## Supporting Information

Figure S1 **PRISMA Flow diagram showing study retrieval and selection.**
(DOCX)

Table S1 Articles describing barrier and immune function of skin in malnourished children.
(DOCX)

Table S2 Articles describing intestinal function and mucosal structure in children with malnutrition.
(DOCX)

Table S3 Articles describing anti-microbial factors in mucosal secretions of malnourished children.
(DOCX)

Table S4 Articles describing commensal flora in children with malnutrition.
(DOCX)

Table S5 Articles describing function of innate immune cells: polymorph-nuclear cells and monocytes/macrophages in children with malnutrition.
(DOCX)

Table S6 Articles describing acute phase response in malnourished children.
(DOCX)

Table S7 Articles describing complement in malnourished children.
(DOCX)

Table S8 Articles describing thymus and other lymphatic tissue in autopsies of malnourished children.
(DOCX)

Table S9 Articles describing ultrasound scans of thymus in malnourished children.
(DOCX)

Table S10 Articles describing delayed type hypersensitivity response in children with malnutrition.
(DOCX)

Table S11 Articles describing lymphocyte subsets in children with malnutrition.
(DOCX)

Table S12 Articles describing antibody levels in children with malnutrition.
(DOCX)

Table S13 Articles describing humoral vaccination responses in children with malnutrition.
(DOCX)

Table S14 Articles describing cytokines in malnourished children.
(DOCX)

Checklist S1 **PRISMA Checklist.**
(DOCX)
