# Supplementary material for: The Immune System in Children with Malnutrition—A Systematic Review
Source: PLoS One. 2014 Aug 25;9(8):e105017. doi: 10.1371/journal.pone.0105017 (PMC4143239; doi:10.1371/journal.pone.0105017)
Supplement: Figure S1 — PRISMA Flow diagram showing study retrieval and selection. (DOCX) [file pone.0105017.s001.docx]

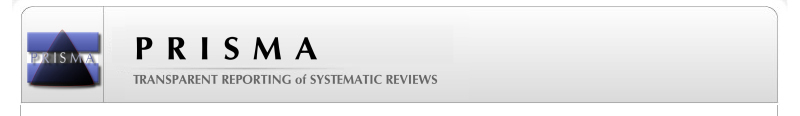
**PRISMA 2009 Flow Diagram**

Records identified through PubMed search
(n = 3402)

Records identified through personal communication

(n=631)

Records identified in reference lists

## Identification

## Screening

## Eligibility

Studies included in qualitative synthesis
(n =245)

Studies of acquired immune system

## Included

Studies of innate immune system

Lymphatic tissue (n=12)

Lymphocytes (n=58)

Acute phase response (n=24)

Complement (n=24)

White blood cells (n=38)

Cytokines (n=35)

Vaccination response (n=35)

Antibody levels (n=32)

Microbial flora (n=11)

Secre-tions
(n =19)

Skin
(n =6)

Gut function
(n =21)
