# Supplementary material for: The Immune System in Children with Malnutrition—A Systematic Review
Source: PLoS One. 2014 Aug 25;9(8):e105017. doi: 10.1371/journal.pone.0105017 (PMC4143239; doi:10.1371/journal.pone.0105017)
Supplement: Table S1 — Articles describing barrier and immune function of skin in malnourished children. (DOCX) [file pone.0105017.s002.docx]

**Table S1: Articles describing barrier and immune function of skin in malnourished children**

| **Author, year** | **Country** | **Age, months** | **Mal-nourished** | **Infec-tions?** | **Cont-rols** | **Infections, con-trols?** | **Skin histology** | **Other** | **Cutaneous infl. response** | **OM vs. NOM** |
| --- | --- | --- | --- | --- | --- | --- | --- | --- | --- | --- |
| **Thavaraj 1989** | India | 12-60 | 5 OM, 12 MK, 3 NOM | yes | 20 | no | Thin stratum granulosum, exageration of stratum corneum | Normal exocrine glands |  | - |
| **Bhaskaram 1982** | India | 12-60 | 15 OM *(WHO)* | no | 10 | no | - | - | Fewer macrophages,  more granulocytes | - |
| **Edelman 1977** | Thailand | 12-60 | 9 OM *(WHO)* | yes | 10 | yes | - | - | More total cells. Fewer macrophages and monocytes, more granulocytes | - |
| **Kulapongs 1977** | Thailand | 12-60 | 9 OM *(WHO)* | yes | 20 | half | - | - | More total cells. Fewer macrophages, more granulocytes | - |
| **Freyre 1973** | Peru | 6-36 | 23 OM, 10 NOM | No | 12 | no | - | - | Fewer monocytes,  more granulocytes in OM. | yes, NOM similar to WN |
| **Sims 1968** | South Africa | ? | 10 OM *(WHO)* | ? | 5 | ? | Thin stratum basale and spinosum. Atrophy of stratum corneum, | Smaller desmosomes |  | - |

Legend: OM=Oedematous malnutrition; MK=Marasmic kwahiorkor; NOM=Non-oedematous malnutrition; *(WHO)=* Children fulfilling WHOs current diagnostic criteria for severe acute malnutrition; WN = well-nourished,;
