# Supplementary material for: The Immune System in Children with Malnutrition—A Systematic Review
Source: PLoS One. 2014 Aug 25;9(8):e105017. doi: 10.1371/journal.pone.0105017 (PMC4143239; doi:10.1371/journal.pone.0105017)
Supplement: Table S2 — Articles describing intestinal function and mucosal structure in children with malnutrition. (DOCX) [file pone.0105017.s003.docx]

**Table S2: Articles describing intestinal function and mucosal structure in children with malnutrition**

| **Author, year** | **Country** | **Age, months** | **No and type of MN** | **Infections, MN** | **WN controls** | **Infections, WN** | **Biopsy from** | **Mucosal thickness** | **Villous height** | **Cellular infiltration** | **Per-meability** | **Other** | **Comments** | **OM vs NOM?** |
| --- | --- | --- | --- | --- | --- | --- | --- | --- | --- | --- | --- | --- | --- | --- |
| **Boaz 2013** | India | 6-59 | 21 UW | 16 diarrhea | 33 | half diarrhea | - | - | - | - | LM-ratio: ↑ | Permeability most affected by diarrhea | - | - |
| **Hos-sain 2010** | Bang-ladesh | 6-24 | 97 severe UW | yes | 17  and ** | no | - | - | - | - | LM-ratio: ↑ | Mannitol uptake most affected in MN | LM-ratio ↑ in MN than WN, and fell after 3 months. No effect of food supplementation | - |
| **Amadi 2009** | Zam-bia |  | 18 NOM,  8 MK,  15 OM | diarrhea | 19 from UK and ** | no | Duodenum | - | ↓/0 | 0/↑ | - | Villous height ↓ , crypts deeper and more lymphocytes than UK children, but recovery: 0 | HLA-DR ↑ and CD3-cells ↑ in lamina propria compared to UK children, but no different that after recovery | HLA-DR, CD3-cells ↓ in OM |
| **Campbell 2003** | Gam-bia | 6-36 | 11 UW,  25 NOM or OM | some diarrhea | 4 local, and 19 from UK | no | Small intestine | - | ↓/0 | 0/↑ | LM ratio: 0 | Villous height ↓ , crypts deeper than UK children, but no diff from WN children form Gambia | CD3 cells and CD25 ↑ in Gambian compared to UK  Anti-inflammatory/ inflammatory cytokine ratio ↓ in MN | - |
| **Mish-ra 2001** | India | 12-60 | 49 UW | diarrhea | 8 | diarrhea | Duodenum | - | 0 | 0 | - | No difference in endoscopic appearance | Histological changes depended on duration of diarrhea | - |
| **Brew-ster 1997** | Malawi | Mean 28 | 149 OM *(WHO)* | some | 45 and ** | ? | - | - | - | - | LM-ratio: ↑ | ↑ young, wasted, with diarrhea, sepsis and in those who died | LM-ratio ↑ in MN than WN, and also higher than after 3 weeks | - |
| **Sulli-van 1992** | Gam-bia | 6-31 | 12 NOM, 7 MK, 1 OM | diarrhea | ** | no | Jejunum | 0 | - | 0 | LM ratio: 0 | No change with rehabilitation | - | - |
| **Beh-rens 1987** | Gam-bia | 1-23 | 42 UW | Some | 15 | ? | - | - | - | - | LM ratio: ↑ | - | Most increased in severely underweight | - |
| **Romer 1983** | Venezuela | ? | 13 UW  7 NOM  4 MK | yes | 9 | yes | Jejunum | - | - | - | - | MN: grade 1 and 2 atrophy  WN: normal and grade 1 atrophy | - | - |
| **Green 1980** | Gam-bia | 4-34 | 8 NOM 6 OM,  9 MK,  2 UW | yes | 20 | yes, diarrhea | Jejunum | - | 0 | - | - | Villous atrophy in 8/18 MN and in 7/17 WN with diarrhea | - | ? |
| **Kas-chula 1979** | South Africa | 4-47 | 7 NOM  3 OM  3 MK | diarrhea | 6 | yes, diarrhea | Duodenem | 0 | ↓ | 0 | - | - | All lower than normal adult values | Most severe in OM |
| **Brun-ser 1976** | Chile | 2-14 | 7NOM | no | 3 | ? | Jejunum | ↓ | ↓ | - | - | - | - | - |
| **Gra-cey 1974** | Indonesia | 7-54 | 4 OM, 3 NOM 14UW | ? | 21 | no | Stomach | - | - | - | - | Gastritis | - | - |
| **Shiner 1973** | South Africa | 12-20 | 5 OM *(WHO)* | yes | ** (only one) | no | Small intestine | - | - | - | - | Villous atrophy; EM: shortened microvilli,  Sparse endoplasmatic reticulum | - | - |
| **Schneider 1972** | Guatemala | 23-61 | 11 OM *(WHO)* | 5 diarrhea | 4 WN and ** | no | Duodenum or jejunum | ↓ | 0 | ↑ | - | - | Ridged villi, even after recovery | - |
| **Red-mond 1971** | South Africa | 6-29 | 20 OM *(WHO)* | ? | ** | no | Rectum | ↓ | - | ↑ | - | Increased vascularity, rectal prolapse, mild atrophy of | Reversible with recovery | - |
| **The-ron 1971** | South Africa | 11-36 | 16 OM *(WHO)* | ? | ** | no | Duodenum or jejunum | ? | ? | ? | - | Lipid droplets in epithelial cells and no chylomicrons between cells | Not stated if improved after recovery | - |
| **Brun-ser 1968** | Chile | 6-30 | 11 OM  18 NOM | ? | 8 | no | Duodenum or jejunum | 0/↓ | 0/↓ | 0/↑ | - | - | Thin mucosa in NOM, not OM  Villous atrophy and cellular infiltration in OM, not NOM | yes |
| **Brun-ser 1966** | Chile | 6-30 | 18 NOM  10 OM | ? | 8 | no | Jejunum | 0/↓ | 0/↓ | 0/↑ | - | - | Thin mucosa in NOM, not OM  Villous atrophy and cellular infiltration in OM, not NOM | yes |
| **Bur-man 1965** | Kenya | 12-36 | 17 OM *(WHO)* | ? | 15 from UK | no | Jejunum | - | ↓ | ↑ | - | Broad villi in ridges | - | - |
| **Stan-field 1965** | Uganda | 12-36 | 21 OM *(WHO)* | ? | 3 | ? | Duodenum or jejunum | - | ↓ | ↑ | - | Villi in ridges  Deeper crypts, shorter villi |  | - |

Abbreviations: NM: Malnourished; WN= well-nourished; NOM= Non-oedematous malnutrition, OM=Oedematous malnutrition, MK= Marasmic-kwashiorkor, defined by both wasting and oedema, UW=Underweight, defined by low weight-for-age; *(WHO)=* Children fulfilling WHOs current diagnostic criteria for severe acute malnutrition; LM= Laktulose-mannitol; *= population of children divided by nutritional status, **malnourished children compared to themselves after nutritional recovery, ↑=higher in malnourished than well-nourished, ↓=lower in malnourished than well-nourished, 0= not different in malnourished and well-nourished; - = not assessed
