# Supplementary material for: The Immune System in Children with Malnutrition—A Systematic Review
Source: PLoS One. 2014 Aug 25;9(8):e105017. doi: 10.1371/journal.pone.0105017 (PMC4143239; doi:10.1371/journal.pone.0105017)
Supplement: Table S3 — Articles describing anti-microbial factors in mucosal secretions of malnourished children. (DOCX) [file pone.0105017.s004.docx]

**Table S3: Articles describing anti-microbial factors in mucosal secretions of malnourished children.**

| **Author, year** | **Country** | **Age, months** | **Number / type of MN** | **Infections, MN?** | **No WN controls** | **Infections, WN?** | **sIgA** | **Secretion studied** | **Other** | **Comments** | **OM vs NOM?** |
| --- | --- | --- | --- | --- | --- | --- | --- | --- | --- | --- | --- |
| **Miller 2012** | Kenya | Mean 10,6 | 42 mod. stu * | ? | 197 | ? | ↑ | Saliva | sIgA in saliva correlated with sIgA in mothers breast milk | Not severely MN | - |
| **Marei 1998** | Egypt | 6-36 | 40, NOM and OM | half diarrhoea | 20 | ? | ↑/0 | Urine | No association between serum IgA and sIgA | sIgA ↑ with diarrhea  no different in MN vs. WN | - |
| **Gilman 1988** | Bangladesh | Mean 40 | 4 NOM, 5 OM, 26 MK | (yes) | 20 | no | - | Gatric juice | Basal secretion:  Acidity: 0; Volume:↓; Gastrin stimulated: Acidity:↓; Volume: ↓ | Bacterial colonization of the gastric mucosa correlated with pH | - |
| **Watson 1985** | Columbia | 18-60 | 39 UW, 21 OM, 11 MK,  11 NOM | yes, in severely MN | 24  ** | no | ↓  ↓/0 | Saliva  Tears | ↓lysozyme in tears, but not saliva of severely MN; Increased flow of saliva after re-nutrition | UW compared to WN: sIgA ↓(as % of total protein); Compared to themselves after recovery: sIgA ↓in saliva but not in tears (as mg/dl) | sIgA saliva ↓ in NOM |
| **Beatty 1983** | South Africa | 9-48 | 4 OM *(WHO)* | yes | ** | no | ↑ | Duodenal biopsy  culture | - | - | - |
| **Yakubu 1982** | Nigeria | ? | 20 OM *(WHO)* | ? | 40 | 20 | ↓ | Nasal washing | Not reduced in WN children with diarrhea | Expressed as % of total protein | - |
| **Green 1980** | Gambia | 4-34 | 8 NOM, 6 OM, 9MK, 2UW | yes | 20 | yes | ↓ | Jejunal biopsy staining for sIgA | - | - | - |
| **Kaschula 1979** | South Africa | 4-47 | 7 NOM  3OM, 3MK | yes, diarrhoea | 6 | yes | 0 | Jejunal biopsy staining for sIgA | - | All lower than normal adult values | ↓ in NOM |
| **Watson 1978** | Colombia | 24-60 | 26 UW | No | 27 | no | ↓ | Tears | ↓lysozyme | sIgA/total protein | - |
| **Ibrahim 1978** | Egypt | 6-36 | 37 NOM, 18 OM, 15 MK | (no) | 15 | no | ↓/↑ | Saliva | Total protein ↑  reduced secretion? | sIgA not standardized for total protein content | Yes, IgA, sIgA and protein ↑OM |
| **McMurray 1977** | Colombia | 18-24 | 12 moderate UW | (no) | 27 |  | ↓  ↓ | Tears  Saliva | Lysozyme: 0 | - |  |
| **Sirisinha 1977** | Thailand | 12-60 | 7 NOM, 9 MK, 8OM | ? | 23 | no | ↓ | Nasal washing | - | - | No |
| **Reddy 1976** | India | 12-60 | 15 OM  15 NOM | half, diarr-hoea | 12 | half | 0/↓ | Duodenal fluid, nasal washing, saliva, tears | - | Reduced in severely MN, both as g/l or as % of total protein. Not reduced in mild-moderate UW | No |
| **Bell 1976** | Indo-nesia | 12-48 | 8 UW, 10 NOM, 3 OM, 8 MK | yes | 10 | yes | 0 | Duodenal fluid | - | 4/10 of control group UW |  |
| **Shashidhar 1976** | India | 12-60 | 15 OM, 15 NOM | ? | 12 | no | - | Gastric juice | Higher pH in MN | - | No |
| **Sirisinha 1975** | Thailand | 12-60 | 7 NOM, 9 MK  8 OM | yes | 23 | no | ↓ | Nasal washing | - | - |  |
| **Gracey 1977** | Indo-nesia | 7-54 | 4 OM, 3 NOM, 14 UW | ? | 21 | no | - | Gastric juice | Acid secretion: ↓, unstimulated and in response to gastrin | Biopsy: gastritis in 8/9 | - |
| **Buchannan 1973** | South Africa | 4-36 | 7 OM and NOM | (no) | 6 | (no) | ↑ | Urine | - | - | - |
| **Adesola 1968** | Nigeria | ? | 15 OM *(WHO)* | ? | 15 | no |  | Gastric juice | Acid secretion: ↓ in response to histamin | - | - |

Legend: MN= malnourished, WN= well-nourished; NOM = non-oedematous malnutrition; OM = oedematous malnutrition; MK = marasmic-kwashiorkor, defined by both wasting and oedema; UW = underweight, defined by low weight-for-age; Stu=stunted, defined by low height-for-age; *(WHO)=* Children fulfilling WHOs current diagnostic criteria for severe acute malnutrition; sIgA= secretory immunoglobulin A;; *= population of children divided by nutritional status; **malnourished children compared to themselves after nutritional recovery; ↑=higher in malnourished than well-nourished; ↓=lower in malnourished than well-nourished; 0= not different in malnourished and well-nourished; - = not assessed; ? = not stated.
