# Supplementary material for: The Immune System in Children with Malnutrition—A Systematic Review
Source: PLoS One. 2014 Aug 25;9(8):e105017. doi: 10.1371/journal.pone.0105017 (PMC4143239; doi:10.1371/journal.pone.0105017)
Supplement: Table S4 — Articles describing commensal flora in children with malnutrition. (DOCX) [file pone.0105017.s005.docx]

**Table S4: Articles describing commensal flora in children with malnutrition.**

| **Author, year** | **Country** | **Age, months** | **No and type of MN** | **Infections, MN?** | **No WN con-trols** | **Infections, WN?** | **Method** | **Media** | **Findings** | **Other** | **Comments** | **OM vs NOM?** |
| --- | --- | --- | --- | --- | --- | --- | --- | --- | --- | --- | --- | --- |
| **Subramanian 2014** | Bangladesh | 6-20 | 61 NOM  3 OM  *(WHO)* | yes | 12 and ** | (no) | sequncing bacterial DNA, model of “microbial maturity” | stool | Immature microbiota pattern in MN, improved with treatment | Relapsed to immature pattern after cessation of treatment |  | - |
| **Smith 2013** | Malawi | 10-16 | 13 twin pairs discordant for OM  *(WHO)* | some | 13 twins to patients and 9 healthy twin pairs | some | sequncing bacterial DNA, PCA | stool | Different pattern of microbiota assessed by PCA in OM compared to healthy | Transplanted microbiota from 3 twin pairs discordant for OM into gnotobiotic mice | More immature pattern = less diversity | - |
| **Monira 2011** | Bangladesh | 24-36 | 7 NOM | no | 7 | no | sequncing bacterial DNA | stool | More proteopacteria and less *Bacteroides, Firmicutes* and *Actinobacteria*. | More *Escherichia, Neisseria* and *Klebsiella* | - | - |
| **Gupta 2011** | India | 16 | 1 ? | ? | 1 | ? | cequncing bacterial DNA | stool | More *campylobacteracae* and *Helicobacteraecea*, less *Lactobacillales, Enterobacteriales, Pseudomonales, Bifidobacteriales* | More proteins encoding for motility, chemotaxis, respiration, and virulence in MN | - | - |
| **Scheutz 1997** | Tanzania | 18-60 | 241 UW* | no | 641* | no | microscopy | mouth swab, | Carriage of yeast more frequent in MN | Even after adjusting for HIV status |  | - |
| **Matee 1995** | Tanzania | 6-24 | 227 UW* | no | 745* | no | microscopy | mouth swab, | Carriage of yeast more frequent in MN | Both wasting and UW ↑ carriage of yeast | - | - |
| **Omoike 1989** | Nigeria | 2-60 | 30 (NOM, OM, MK) | 19 diarrhea | 22 | half diarrhea | culture | duodenal aspirate and throat swaps | Duodenum: More bacteria in MN than WN, irrespective of diarrhea  Throat: more candida | In MN: Yeast, Kleibsiella, E.Coli.  In WN: gram positive cocci | - | No |
| **Gilman 1988** | Bangladesh | Mean 40 | 4 NOM, 5 OM, 26 MK | (yes) | 20 | no | culture | gastric aspirate | 25/33 of MN colonized with g.neg bacteria, none of the WN | Many of the WN also had high gastric pH, despite this not colonized | - | - |
| **Neto 1976** | Brazil | 1-18 | 22 UW | diarrhoea | 16 | 5 with diarrhea | culture | duodenal aspirate | Bacteria present in those with diarrhea, both MN and WN | - | No MN without diarrhea | - |
| **Gracey 1974** | Autralia/ Indonesia | ? | 57 most MN | yes | 27, most WN | some | culture | gastric and small intestinal aspirates | Candida more frequent in predominantly malnourished children | Peculiar design | - | - |
| **Mata 1972** | Guatemala | 12-60 | 13 (NOM and OM) | most diarrhea | 4 and ** | no | culture | Gastric and small intestinal aspirate, stool | In MN: Same amount of bacteria in stomach, more in small intestine | In WN: More bacteria in small intestine than expected. Reduced bacteria with nutritional rehabilitation | - | - |

Legend: MN= malnourished, WN= well-nourished, NOM= non-oedematous malnutrition, OM= Oedematous malnutrition, MK= Marasmic-kwashiorkor, defined by both wasting and oedema; UW=Underweight, defined by low weight-for-age, Stu=stunted, defined by low height-for-age; *(WHO)=* Children fulfilling WHOs current diagnostic criteria for severe acute malnutrition; PCA= Principal component analysis.*= population of children divided by nutritional status, **malnourished children compared to themselves after nutritional recovery, ↑=higher in malnourished than well-nourished, ↓=lower in malnourished than well-nourished, 0= not different in malnourisched and well-nourished; - = not assessed; ? = not reported.
