# Supplementary material for: The Immune System in Children with Malnutrition—A Systematic Review
Source: PLoS One. 2014 Aug 25;9(8):e105017. doi: 10.1371/journal.pone.0105017 (PMC4143239; doi:10.1371/journal.pone.0105017)
Supplement: Table S5 — Articles describing function of innate immune cells: polymorph-nuclear cells and monocytes/macrophages in children with malnutrition. (DOCX) [file pone.0105017.s006.docx]

**Table S5: Articles describing function of innate immune cells: granulocytes and monocytes/macrophages in children with malnutrition.**

| **Author, year** | **Country** | **Age, months** | **No MN** | **Infections, MN?** | **WN controls** | **Infections, WN?** | **Total leukocytes** | **Cells** | **Phagocytosis** | **Metabolism phagocytosis** | **Chemotaxis/motility** | **Microbicidal activity** | **H202 production** | **Other** | **OM vs. NOM** |
| --- | --- | --- | --- | --- | --- | --- | --- | --- | --- | --- | --- | --- | --- | --- | --- |
| **Hughes 2009** | Zambia | 12-60 | 24 NOM, 57 OM | yes | ** | no | ↑ | N | - | - | - | - | - | Lower levels of dendritic cells in MN  Lower T-cell IFNγ response to recall tuberculin | ? |
| **Nassar 2009** | Egypt | Mean 11 | 18 NOM, 12 OM | ? | 12 and ** | ? | ↑ | Mon  N | - | - | - | - | - | More monocytes and neutrophils with marker of apoptosis (CD95) in MN. | 0 |
| **Nassar 2007** | Egypt | Mean 12 | 18 NOM  14 OM | ? | 14 | no | ↑ | - |  |  |  |  |  |  |  |
| **Nájera**  **2007** | Mexico | 7-29 | 3 NOM, 2 OM, 3 UW | yes | 21 | 10 of them | 0 | - |  |  |  |  |  |  |  |
| **Nájera 2004** | Mexico | 8-29 | 7 NOM, 3 OM, 5 UW | yes | 22 | 12 of them | 0 | - |  |  |  |  |  |  |  |
| **Vasquez-Garibay 2004** | Mexico | 3-18 | 12 NOM | no | ** | - | - | N  GRAN | ↓ | - | ↓ | ↓ | - | Same population as study below? | - |
| **Vasquez-Garibay 2002** | Mexico | 3-18 | 12 NOM | no | ** | - | - | N  GRAN | ↓ | - | ↓ | ↓ | - |  | - |
| **Gonzales 2002** | Mexico | 6-29 | 5 NOM  1 OM | yes | 12 | half | - |  |  |  |  |  | - | ↑leucocytes with DNA damage, ↑ H2O2 induced DNA damage, compared to WN w/wo inf. Less DNA repair capacity in MN |  |
| **Gonzales 2002** | Mexico | 6-29 | 6 NOM | yes | 6 | yes | - |  |  |  |  |  | - | ↑leucocytes with DNA damage and higher increase in DNA damage after 1 week of antibiotics |  |
| **Nájera 2001** | Mexico | 6-24 | 6 MAM, 6 NOM, 3 OM | yes | 22 | 12 of them | 0 |  |  |  |  |  |  |  |  |
| **Fongwo 1999** | Nigeria | ? | 41 NOM, 19 OM, 5 MK | ? | 35 | ? | 0 | Leu | - | - | - | - | - |  | 0 |
| **Rikimaru 1998** | Ghana | 8-36 | 49 UW, 28 OM, 27 NOM | (no) | 61 | no | - | NK | - | - | - | - | - | No difference in NK cells | 0 |
| **Lotfy 1998** | Egypt | 5-20 | 12 NOM, 16 OM, 12 MK | yes | 30 | no | ↑ | Mon | ↓ | - | - | ↓ | - |  | Most ↓ in OM |
| **Raman 1992** | India | ? | 28 NOM, 22 OM | no | 25 | no | - | GRAN | - | - | - | - | ↓ |  | ↓ in OM |
| **Chhangani 1985** | India | ? | 60 (UW, OM, MK, NOM) | ? | 10 | ? | - | GRAN | - | - | - | ↓ | - |  | ↓ in OM |
| **Machado 1985** | Brazil | 12-45 | 7 OM,  4 MK | yes | 8 | no | - | GRAN | - | - | - | - | ↑ |  | - |
| **Forte 1984** | Brazil | 5-60 | 20 UW | no | 40 | no | - | Mon | ↓ | - | - | - | ↓ |  | - |
| **Salimonu 1983** | Nigeria | ? | 8 OM  15 NOM | (no) | 20 | no | - | NK | - | - | - | - | - | No diff. in mean NK cell avtivty. More MN children with very low NK cell activity. Less response to IFN than well-nourished. Normalisation with nutritional rehabilitation | 0 |
| **Salimonu 1982** | Nigeria | 12-60 | 5 OM  14 NOM | (no) | 16 | ? | - |  | - | - | - | - | - | Mean activity/number of NK cells: 0. More MN with very low NK counts. Less increase w stimulation w interferon | - |
| **Bhaskaram 1982** | India | 24-60 | 6 OM *(WHO)* | no | 9 | no | - | Mon | - | - | - | 0 | - | Monocytes transformed to macrophages:0 | - |
| **Goyal 1981** | India | 12-60 | 25 NOM | no | 25 | no | - | PNMC | - | - | - | - | - | ↓ adherence | - |
| **Bhaskaram 1980** | India | 25-60 | 11 OM *(WHO)* | no | 9 | no | - | Mon | - | - | - | 0 | - | Transformation of monocytes to macrophages: 0 | - |
| **Keusch 1977** | Guate-mala | Mean 36 | 5 OM, 7 MK, 2 NOM | yes | 3 | yes | 0 |  |  |  |  |  |  | EM: Frequent plasmacytoid cells |  |
| **Rich 1977** | Ghana | 12-48 | 13 severe MN | no | 13 | no | - | Neu | - | - | 0 | - | - |  | - |
| **Leitzmann 1977** | Thailand | 12-60 | 16 NOM, 14 MK, 12 OM | yes | ? | no | - | PNMC | 0 | - | - | - | - | Controls healthy adults | ? |
| **Keusch 1977** | Guatemala | Mean 42 | 6 NOM, 7 UW | yes | 6 | yes | 0 | Leu  GRAN | - | 0 | - | 0 | - | All values lower than in non-infected children | - |
| **Reddy 1976** | India | 12-60 | 15 severe MN, 9 UW | ? | 15 | ? | - | Leu | - | ↓ | - | - | - | ↓ CO2 production. 0 lactate production | - |
| **Shilotri 1976** | India | 12-60 | 11 ? | ? | 8 | no | - | GRAN | - | - | - | - | ↓ | Less increase with phagocytosis in MN | - |
| **Schopfe 1976** | Ivory Coast | Mean 21 | 46 OM *(WHO)* | no | 40 | ? | 0 | N | 0 | 0 | ↓ | ↓ | ↑ | more eosinophils, immature GRAN, prominent endoplasmatic reticulum | - |
| **Schopfer 1976** | Cote d’Ivoire | ? | 72 OM  *(WHO)* | no | 36 | no | 0 | GRAN | 0 | - | - | - | - | EM: More neutrophils, Pleomorphism of granulae. Immature GRAN | - |
| **Purtilo 1976** | Brazil | 15-50 | 3 OM, 5 NOM, 41 UW* | yes | 14 | yes | 0 |  |  |  |  |  |  |  |  |
| **Rosen 1975** | South Africa | 6-24 | 35 OM *(WHO)* | some | 21 | some | 0 | PNMC | - | - | 0 | 0 | - | most affected by infections – not by malnutrition | - |
| **Shousha 1974** | Egypt | 12-36 | 37 OM *(WHO)* | yes | ** | no | - | Leu | - | - | - | - | - | Higher levels of intracellular enzymes: alkaline phosphatase, acid phosphatase, myeloperoxidase, lactic dehydrogenase | - |
| **Wolfsdorf 1974** | South Africa | Mean 17 | 50 OM *(WHO)* | half | 25 | no | - | N | - | - | - | - | ↑ | In OM both with and without infections | - |
| **Douglas 1974** | Ivory Coast | 10-30 | 16 OM *(WHO)* | no | 16 | ? | - | GRAN  Mon | 0 | 0 | - | ↓ | - |  | - |
| **Shousha 1972** | Egypt | 12-36 | 14 OM *(WHO)* | yes | 13 | no | - | N | 0 | 0 | 0 |  | ↓ |  | - |
| **Altay 1972** | Turkey | 5-96 | 25 UW | no | 15 | no | - | GRAN | - | - | - | - | 0 |  | - |
| **Tejada 1964** | Guatemala | 36-60 | 8 OM  *(WHO)* | yes | 12 | no | - | N | 0 | - | - | - | - | Higher alkaline phosphatase in neutrophils in OM | - |

MN= malnourished, WN= well-nourished; NOM = non-oedematous malnutrition, OM = Oedematous malnutrition, MK= marasmic kwashiorkor, defined by both wasting and oedema; UW=Underweight (low weight-for-age); *(WHO)=* Children fulfilling WHOs current disgnostic criteria for severe acute malnutrition; IFNγ= interferron gamma; PHA= phytohemaglutinin; N= neutrophils; GRAN= granulocytes (poly-morphnuclear cells); Mon= monocytes (sometimes transformed in vitro to macrophages); Leu= leucocytes; NK= Natural killer; HMS= Hexose Monophosphate shunt; EM: Electron microscopy;↓=lower in malnourished than well nourished, ↑ = higher in malnourished than well-nourished , 0= not different in malnourished and well nourished, ** malnourished children compared to themselves after recovery; - = not assessed;
