# Supplementary material for: The Immune System in Children with Malnutrition—A Systematic Review
Source: PLoS One. 2014 Aug 25;9(8):e105017. doi: 10.1371/journal.pone.0105017 (PMC4143239; doi:10.1371/journal.pone.0105017)
Supplement: Table S6 — Articles describing acute phase response in malnourished children. (DOCX) [file pone.0105017.s007.docx]

**Table S6: Articles describing acute phase response in malnourished children.**

|  | | | | | | |  | **Positive Acute Phase Proteins** | | | | | **Negative Acute phase proteins** | | |  | | |
| --- | --- | --- | --- | --- | --- | --- | --- | --- | --- | --- | --- | --- | --- | --- | --- | --- | --- | --- |
| **Author, Year** | **Country** | **Age, months** | **Malnourished** | **Infections** | **WN** | **Infections, WN** | **Fever** | **CRP** | **AA** | **HG** | **CP** | **α 1AT** | **TF** | **α2-HS-G** | **PA** | **Other** | **Comments** | **NOM vs. OM?** |
| **El-Sayed 2006** | Egypt | 3-36 | 16 NOM  14 OM | yes | 10 | ? | - | ↑ | - | - | - | - | - | - | - |  | - | 0 |
| **Manary 2004** | Malawi | 12-60 | 25 NOM | yes | 13 | yes | - | ↓ | 0 | 0 | - | 0 | - | - | - | CRP lower despite higher IL6 | - | - |
| **Reid 2002** | Jamaica | Mean 11 | 9 NOM, 14 OM | yes | ** | - | - | ↑ | ↑ | ↑ | - | ↑ | - | - | - | IL6 ↑  fibrinogen: 0 | - | yes, α1-AT and HG ↑ in NOM  CRP,IL6,AA: 0 |
| **Morlese 1998** | Jamaica | Mean 10 | 9 NOM | yes | ** | - | - | ↑ | ↑ | ↑ |  | ↑ | - | - | - | fibrinogen: 0 | Same as above? | - |
| **Malavé 1998** | Venezuela | 6-60 | 46 UW | half | 61 | half | - | 0 | - | - | - | - | - | - | ↓ | CRP and IL6 rise with infection: 0; PA ↓ in MN without infections | - | - |
| **Hassanein 1998** | Egypt | 6-36 | 15 moderate UW | no | 10 | no | - | - | - | - | - | - | ↓ | - | - | fibronectin: ↓ | - | - |
| **Rikimaru 1998** | Ghana | 8-36 | 28 OM, 27 NOM, 39 UW | (no) | 61 | (no) | - | ↑ | - | - | - | - | ↓ | - | - | - | - | No |
| **Sauerwein 1997** | Kenya | Mean 37 and 30 | 30 OM, 16 NOM | some | 39 | some | - | ↑ | - | - | - | - | - | - | - | - | - | CRP ↑ in OM |
| **Akenami 1997** | Nigeria | Mean 2,2 | 20 NOM, 20 OM, 20 MK | yes | 20 | no | - | - | - | - | - | - | ↓ | - | - | fibronectin ↓ | - | yes: TF ↓ in OM, fibronectin: 0 |
| **Ekanem 1997** | Nigeria | 3-60 | 14 OM, 7 NOM, 6 MK | 17 | 10 | no | - | ↑ | - | - | - | - | - | - | - | CRP ↑ with no infections. Normal CRP rise in infection | cutoff of 20mg/l | - |
| **Doherty 1993** | Jamaica | 6-36 | 21 NOM, 18 OM, 21MK, 6 UW | 50 | 10 | no | - | 0 | - | - | - | - | - | - | - | serum-amyloid-A: 0;  response to DTP vaccination: 0 from WN, but ↑at 2nd dose after rehabilitation | - | 0 |
| **Dao 1992** | Mali | 8-22 | 200* | no | * | ? | - | - | - | - | - | - | - | - | ↓ | PA correlated with W/H and W/A | - | - |
| **Doherty 1989** | Jamaica | 6-25 | 4 NOM, 3 OM, 8 MK, 1 UW | (no) | ** | no | ↓ | - | - | - | - | - | - | - | - |  | Response to 1^st^ and 2^nd^ DTP vaccine | - |
| **Abiodun 1987** | Nigeria | 6-36 | 17 NOM, 22 OM | half | 32 | half | - | - | - | - | - | - | - | ↓ | - |  | ↓ with infection | lowest values in infected OM |
| **Yoder 1987** | USA | Mean 2,7 | 20, low MUAC | no | 20 | no | - | - | - | - | - | - | - | - | ↓ | fibronectin ↓ | - | - |
| **Abiodun 1985** | Nigeria | 6-36 | 14 OM, 10 NOM | (no) | 16 | no | - | - | - | - | - | - | - | ↓ | - |  | - | 0 |
| **Salimonu 1985** | Nigeria | 12-48 | 58 NOM, 13 OM | (no) | 22 | ? | - | ↑ | - | - | - | ↑/0 | - | - | - | α1-AT ↑ in NOM, not in OM; α-2M ↓ | - | α1-AT ↑, α-2M ↓ in NOM |
| **Idris 1983** | Sudan | 13-36 | 23 NOM, 12 MK | ? | 35 | ? | ↑ | - | - | - | - | - | - | - | - | reactions: Rash ↓  Fever, pneum, diarrhea ↑, | measles vaccination | ? |
| **Schelp 1980** | Thailand | 7-60 | 6 NOM, 7 OM | yes | 6 | no | - | - | - | - | - | - | - | ↓ | - |  | - | ? |
| **McMurray 1979** | Colombia | 10 | 6 UW | (no) | 30 | (no) | 0 | - | - | - | - | - | - | - | - | after measles vaccine | - | - |
| **McFarlane 1977** | Nigeria | 12 – 60 | 75 OM  *(WHO)* | ? | 30 | ? | - | ↑ | - | ↑ | - | ↓ | ↓ | - | - | α-2M ↓ | - | - |
| **Nahani 1976** | Iran | 4-38 | 24 NOM, 29 UW* | no | 35 | no | - | - | - | - | ↓ | - | ↓ | - | - | - | - | - |
| **Razban 1975** | Nigeria | 12-60 | 50 OM, 25 NOM | ? | 30 | ? | - | ↑ | - | ↑ | ↑ | ↓ | - | - | - | - | - | - |
| **Parent 1974** | South Africa | 8-72 | 32 N/OM | ? | ** | - | - | - | - | - | - | - | ↓ | - | - | - | - | - |

Abbreviations: MN = Malnourished; WN = Well-nourished; NOM = non-oedematous malnutrition; OM= oedematous malnutrition; MK= Marasmic-kwashiorkor, defined by both wasting and oedema; UW=Underweight, defined by low weight-for-age; Stu=stunted, defined by low height-for-age; *(WHO)=* Children fulfilling WHOs current disgnostic criteria for severe acute malnutrition; WN=well-nourished; *= population of children divided by nutritional status, **malnourished children compared to themselves after nutritional recovery; ↑=higher in malnourished than well-nourished, ↓=lower in malnourished than well-nourished, 0= not different in malnourished and well-nourished; CRP= C-reactive Protein; AA=α_1_-acid glycoprotein, also known as orosomucoid; HG= haptoglobin; CP = caeruloplasmin; α1-AT= α-1-antitrypsin; TF= Transferrin; α2-HS-G: α2-HS-glycoprotein, also known as fetuin-A; PA= Pre-albumin, also known as trans-thyrein; α-2M= alpha-2-macroglobulin; IL-6 = Interleukin-6; DTP = Diphteria-Tetanus-Pertussis; W/A = weight-for-age; W/H = Weight-for-height;
