# Supplementary material for: The Immune System in Children with Malnutrition—A Systematic Review
Source: PLoS One. 2014 Aug 25;9(8):e105017. doi: 10.1371/journal.pone.0105017 (PMC4143239; doi:10.1371/journal.pone.0105017)
Supplement: Table S7 — Articles describing complement in malnourished children. (DOCX) [file pone.0105017.s008.docx]

**Table S7: Articles describing complement in malnourished children.**

| **Author, year** | **Country** | **Age, months** | **Malnourished** | **Infections, MN?** | **No WN** | **Infections, WN?** | **C1** | **C3** | **C4** | **C5** | **C6** | **C9** | **Factor B** | **CH-50** | **ACH-50** | **Other** | **Comments** | **OM vs NOM?** |
| --- | --- | --- | --- | --- | --- | --- | --- | --- | --- | --- | --- | --- | --- | --- | --- | --- | --- | --- |
| **Abdulrhman 2011** | Egypt | 12,3 (mean) | 30 NOM/UW | (no) | 20 | no | - | - | - | - | - | - |  | ↓ | - | - | Better recovery of CH50 with honey | - |
| **Manary 2004** | Malawi | 12-60 | 25 NOM | yes | 13 | yes | - | ↓ | - | - | - | - | ↓ | - | - | - | Despite higher cytokines in MN | - |
| **Rikimaru 1998** | Ghana | 8-36 | 49 UW, 28 OM, 27 NOM | (no) | 61 | no | - | ↓ | ↑ | - | - | - |  | - | - | - | C3 correlated with albumin | 0 |
| **Lotfy 1999** | Egypt | 5-20 | 12 NOM, 12 MK,  16 OM | some | 20 | no | - | 0/↓ | 0/↓ | - | - | - | - | - | - | - | Only ↓ in OM and MK | Yes |
| **Ekanem 1997** | Nigeria | 3-60 | 14 OM,  27 NOM,  6 MK | some | 10 | no | - | ↓ | - | - | - | - | - | - | - | - | C3 rose with infection | - |
| **Ozkan 1993** | Turkey | 3-24 | 29 UW | yes | 15 | yes | - | ↓ | - | - | - | - | - | - | - | - | - | - |
| **Sakamoto 1992** | Guate-mala | ? | NOM | yes | ** | ? | 0 | ↓ | 0 | 0 | - | 0 | - | 0 | ↓ | - | - | - |
| **Forte 1992** | Brazil | 24-60 | 20 UW,  20 NOM | (no) | 20 | (no) | - | 0 | 0 | - | - | - | - | 0 | - | - | - | - |
| **Salimonu 1985** | Nigeria | 12-48 | 58 NOM,  13 OM | ? | 22 | ? | - | ↓ | - | - | - | - | - | - | - | - |  | - |
| **Forte 1984** | Brazil | 6-60 | 20 UW | (no) | 40 | no |  | 0 | 0 |  | - |  | - | 0 | - | - | - | - |
| **Kumar 1984** | India | 12-60 | 19 NOM,  13 OM | yes | 12 | no | - | ↓ | - | - | - | - | - | 0 | 0 | - | - | Yes, C3↓ in OM |
| **Keusch 1984** | Guate-mala | 19-60 | 7 OM *(WHO)* | yes | ** | no | - | - | - | - | - | - | - | ↓ | ↓ | opsonic activity of plasma ↓ | - | - |
| **Beatty 1978** | South Africa | 10-48 | 12 OM *(WHO)* | minor | 10 | no | - | ↓ | - | - | - | - | - | - | - | - | - | - |
| **Haller 1978** | Ivory Coast | 15-25 | 10 NOM,  18 MK,  31 OM | yes | 20 | 10 | - | ↓ | 0 | 0 | - | ↓ | ↓ | ↓ | - | C3d ↑  Cholinesterase ↓ | C3 correlates with albumin | Yes  CH50, C9 ↓  C3d/C3 ↑ |
| **Kielmann 1977** | India | 0 - 24 | 15 NOM,  19 UW | no | 19 | no | - | ↓ | - | - | - | - | - | - | - |  |  | - |
| **McFarlane 1977** | Nigeria | 12 - 60 | 75 OM *(WHO)* | ? | 30 | ? | - | ↓ | - | - | - | - | - | - | - | - |  | - |
| **Rich 1977** | Ghana | 12-48 | 13 OM/NOM | no | 13 | no | - | 0 | 0 | - | - | - | - | - | - |  |  | ? |
| **Hafez 1977** | Egypt | 11-23 | 45 OM, 38 NOM | ? | 30 | ? | - | ↓ | 0/↓ | 0/↓ | 0/↓ | 0/↓ | 0/↓ | - | - | C8, C19, C3PA ↓in OM | All ↓ in OM , only C3 ↓in NOM | only ↓ C3 in NOM |
| **Nahani 1976** | Iran | 4-38 | 24 NOM,  29 UW* | (no) | 35 | no | - | 0 | - | - | - | - | - | - | - | - |  | - |
| **Suskind 1976** | Thailand | 12-60 | 4 NOM, 10 MK, 14 OM | yes | 25 | 8 | - | - | - | - | - | - | - | ↓/ 0 | - | Anti-complement activity ↑ |  | yes, only ↓ in OM |
| **Olusi 1976** | Nigeria | 12-60 | 35 OM, 35 NOM | yes | ? | ? |  | ↓ | 0 | ↑ | ↓ | ↓ |  | - | - | C1q ↓ , C1s ↓ | C3↓ and C5 ↑ with ↑CRP | yes, all  ↓ in OM |
| **Olusi 1975** | Nigeria | 12-60 | 50 OM, 25 NOM | ? | 35 | yes | - | ↓ | 0 | - | - | - | - | - | - | - |  | yes: C3 ↓ in OM |
| **Razban 1975** | Nigeria | 12-60 | 50 OM, 25 NOM | ? | 30 | ? | - | ↓ | - | - | - | - | - | - | - | - | - | - |
| **Sirisinha 1973** | Thailand | 12-60 | 10 NOM, 10 OM | yes | 19 | no | - | ↓ | 0 | ↓ | ↓ | ↓ | ↓ | - | - | C3PA ↓; C1q ↓; C1s ↓ |  | Yes: C1q, C6, C8 ↓ |

Legend: MN = malnourished; WN = Well-nourished; NOM= non-oedematous malnutrition, OM= Oedematous malnutrition, MK= Marasmic-kwashiorkor, defined by both wasting and oedema, UW=Underweight, defined by low weight-for-age; *(WHO)=* Children fulfilling WHOs current diagnostic criteria for severe acute malnutrition; *= population of children divided by nutritional status; **malnourished children compared to themselves after nutritional recovery; ↑=higher in malnourished than well-nourished; ↓=lower in malnourished than well-nourished; 0= not different in malnourished and well-nourished; C = Complement factor; CRP= C-Reactive Protein, Factor B, also known as Complement 3 Pro-activator (C3PA); CH-50= Complement activity of the classical pathway; ACH-50= Complement activity by the alternative pathway; *(WHO)=* Children fulfilling WHOs current disgnostic criteria for severe acute malnutrition
