# Supplementary material for: The Immune System in Children with Malnutrition—A Systematic Review
Source: PLoS One. 2014 Aug 25;9(8):e105017. doi: 10.1371/journal.pone.0105017 (PMC4143239; doi:10.1371/journal.pone.0105017)
Supplement: Table S8 — Articles describing thymus and other lymphatic tissue in autopsies of malnourished children. (DOCX) [file pone.0105017.s009.docx]

**Table S8: Articles describing thymus and other lymphatic tissue in autopsies of malnourished children.**

| **Author, year** | **Country** | **Age** | **MN** | **Infec-tions, MN?** | **WN Controls** | **Infections, WN?** | **Macroscopic appearance** | **Histology Thymus** | **Other lymphatic tissue** | **Other** | **OM vs. NOM** |
| --- | --- | --- | --- | --- | --- | --- | --- | --- | --- | --- | --- |
| **Jambon 1988** | Senegal | 12-48 | 15 NOM,  25 OM | yes | 18 UW | yes | Severe atrophy MN, some atrophy in UW | Increased connective tissue; few lymphocytes; loss of cortico-medullar differentiation; few Hassall corpuscles; few thymulin positive cells | - | Lower thymulin-concentration  Normal zinc concentration | no |
| **Aref 1982** | Egypt | 6-31 | 33 OM,  12 NOM | yes | 15 | yes | Thymic atrophy | Few lymphocytes. Loss of cortico-medullar differentiation. Fibrous tissue and fat. | Smaller spleen, tonsils, LN. Hypocellulær bonemarrow, with fewer plasmacells | Smaller adrenal gland | Yes – lowest thymus weight % in OM |
| **Schonland 1972** | South Africa | 4-47 | 21 UW,  23 NOM,  47 OM | yes | 27 | yes | Low absolute and relative weight | Small lobules; few lymphocytes; loss of cortico-medullar differentiation | Smaller spleen, LN, appendix, Peyers playes. lymphocyte depletion; fewer germinal centers. |  | Similar degree pof atrophy – lowest thymus weight % in OM |
| **Smythe 1971** | South Africa | 4-47 | 23 NOM, 47 OM | ? | 27 | ? | Small thymus | Acute or chronic involution, depletion of lymphocytes, loss of cortico-medullar differentiation | Reduced size of tonsils, peyers plaques, appendix; lymphocyte depletion; few germinal centres |  | Similar thymus atrophy; OM: most abnormal histology |
| **Watts 1969** | Uganda | 0-60 | 22 OM, 26 NOM | yes | 84 | few | Low absolute and relative weight | - | - |  | Most atrophy in OM |
| **Naeye 1956** | USA | 1-5 | 7 ? | ? | 14 | some | Atrophy | Cortex most reduced. | Spleen also reduced in size, to a less extend |  | - |

Abbreviations: MN= malnourished; WM = Well-nourished; NOM= non-oedematous malnutrition; OM= oedematous malnutrition; UW= under-weight; LN= lymph nodes.
