# Supplementary material for: The Immune System in Children with Malnutrition—A Systematic Review
Source: PLoS One. 2014 Aug 25;9(8):e105017. doi: 10.1371/journal.pone.0105017 (PMC4143239; doi:10.1371/journal.pone.0105017)
Supplement: Table S9 — Articles describing ultrasound scans of thymus in malnourished children. (DOCX) [file pone.0105017.s010.docx]

**Table S9: Articles describing ultrasound scans of thymus in malnourished children.**

| **Author, year** | **Country** | **Age, months** | **MN** | **Infec-tions, MN?** | **WN controls** | **Infec-tions, WN?** | **Method** | **Thymus size** | **Other** | **OM vs. NOM** |
| --- | --- | --- | --- | --- | --- | --- | --- | --- | --- | --- |
| **Moore 2009** | Bangladesh | 1-12 | 2094 total* | some | * | no | Ultrasound, thymic index | Thymus size correlated with body weight at fixed ages | Variation by season | - |
| **Garly 2008** | Guinea Bissau | 6 | 923 total * | some | * | some | Ultrasound, thymic index | Thymus size smaller with low W/A, low W/H, low MUAC and in sick children. | Better correlated with W/A and MUAC than with W/H | - |
| **Nassar 2007** | Egypt | Mean 12 | 14 OM, 18 NOM | ? | 14 | no | Ultrasound, thymic diameter in two dimensions, multiplied | Small thymus  Increase with re-nutrition, but not to control values |  | Smallest thymus in OM |
| **Collison 2003** | Gambia | 0-12 | 138 total* | some | * | some | Ultrasound, thymic index | Thymus size correlated with body weight at fixed ages |  | - |
| **Chevalier 1998** | Bolivia | Mean 17 | 92 ** | ? | ** | ? | Ultrasound, thymus area | Thymus smaller in MN  Anthropometric recovery before thymus recovery | Thymus size followed MUAC more than W/H | ? |
| **Chevalier 1997** | Bolivia | Pre-school | 42 NOM and OM | ? | 15 and ** | no | Ultrasound, thymic area | Thymus smaller in MN  Anthropometric recovery before thymus recovery. Zinc supplement accelerated thymus recovery. | Same children as below? | ? |
| **Chevalier 1994** | Bolivia | ? | 42 ? | ? | 15 and ** | ? | Ultrasound, thymic area | Thymus smaller in MN  Anthropometric recovery before thymus recovery. Zinc supplement accelerated thymus recovery. |  | ? |
| **Parent 1994** | Bolivia | 11-28 | 13 NOM  29 OM | ? | 15 | no | Ultrasound, thymic area | Thymus smaller in MN |  | ? |

Legend: MN= malnourished; WN= well-nourished; NOM: non-oedematous malnutrition, OM: oedematous malnutrition; W/A= weight-for-age; W/H= weight-for-height; MUAC: mid-upper arm circumference; * group of children divided by nutritional status; **=compared to themselves after recovery
