# Supplementary material for: The Immune System in Children with Malnutrition—A Systematic Review
Source: PLoS One. 2014 Aug 25;9(8):e105017. doi: 10.1371/journal.pone.0105017 (PMC4143239; doi:10.1371/journal.pone.0105017)
Supplement: Table S10 — Articles describing delayed type hypersensitivity response in children with malnutrition. (DOCX) [file pone.0105017.s011.docx]

**Table S10: Articles describing delayed type hypersensitivity response in children with malnutrition.**

| **Author, year** | **Country** | **Age, months** | **MN** | **Infections, MN?** | **WN** | **Infections, WN** | **Mantoux after vaccination** | **Mantoux unclear vaccination** | **PHA** | **Candida** | **DNCB** | **Other** | **Comments** | **OM vs. NOM?** |
| --- | --- | --- | --- | --- | --- | --- | --- | --- | --- | --- | --- | --- | --- | --- |
| **Sakamoto 1992** | Guate-mala | ? | 8 NOM | yes | ** | no | - | - | ↓ | - | - | - |  | - |
| **Fakhir 1989** | India | 5-60 | 22 UW, 54 NOM, 24 OM | ? | 25 | ? | - | - | - | - | ↓ | - |  | Yes, most ↓ in OM |
| **Greenwood 1986** | Nigeria | 12-24 | 40 UW* | some | 32 | some | 0 | - | - | - | - | - | UW, not SAM | - |
| **Bhaskaram 1982** | India | 12-60 | 15 OM *(WHO)* | no | 10 | no | - | - | ↓ | - | - | - |  | - |
| **McMurray 1981** | Colombia | 18-60 | 22 OM, 11 NOM | yes | 25 | no | ↓ | ↓ | - | ↓ | ↓ | - |  | yes, most ↓ in OM |
| **McMurray 1981** | Colombia | 2-24 | * total 71 | ? | * | ? | ↓ | - | - | - | ↓ | - | Cohort followed 2 years | - |
| **Seth 1981** | India | Pre-school | 93 UW* | ? | 42* | ? | ↓ | - | - | - | - | - |  | - |
| **Satyanarayana 1980** | India | 12-60 | * total 261  9 OM | ? | * | ? | UW: 0  OM: ↓ | - | - | - | - | - |  | yes, most ↓ in OM |
| **Puri 1980** | India | 6-60 | 9 NOM, 5 OM, 8 MK | most | 13 | half | - | ↓ | ↓ | - | - | - |  | no |
| **Kielmann 1977** | India | 0-24 | 51 NOM  83 UW | no | 60 | no | NOM↓  UW:0 | - | - | - | - | - |  | - |
| **Heyworth 1977** | Gambia | 6-40 | *16 NOM,  94 UW | ? | 57* | ? | NOM ↓,  UW 0 | - | - | - | - | - | 6 weeks after vaccination: ↓  26 weeks after: 0 | - |
| **Smith 1977** | Tunesia | 3 – 18 | 39 NOM, 29 MK | yes | 58 | no | ↓ | - | ↓ | ↓ | ↓ | - |  |  |
| **Schlesinger 1977** | Chile | 3-18 | 22 NOM | some | 60 | 8 of them | - | ↓ | - | - | ↓ | - | ↓ in WN infected | - |
| **Ziegler 1975** | Nepal | 10-24 | 24 (W/H)* | no | 158* | no | - | ↓ | - | - | - | - |  | - |
| **Abbassy 1974** | Egypt | 4-40 | 37 OM, 21 NOM | ? | 13 | no | ↓ | - | - | - | - | - |  | yes, most ↓ in NOM |
| **Bhaskaram 1974** | India | 12-60 | 8 UW, 14 OM, 8 NOM | ? | 11 | ? | - | . | ↓ | - | - | - | Only ↓ in OM and NOM | no |
| **Schlesinger 1974** | Chile | 3-18 | 13 NOM | no | 19 | no | - | - | - | - | ↓ | - |  | - |
| **Edelman 1973** | Thailand | 12-60 | 30 NOM, OM | yes | ** | no | - | - | - | ↓ | ↓ | - |  | - |
| **Smythe 1971** | South Africa | ? | 17 OM/NOM | ? | 19 | ? | - | - | - | - | ↓ | - |  | ? |
| **Geefhuysen 1971** | South Africa | 6-30 | 18 OM *(WHO)* | yes | 9 | yes | - | - | - | ↓ | - | diphteria | ↓ | - |
| **Harland 1965** | Uganda | ? | 30 UW | ? | 17 | ? | ↓ | - | - | - | - | - |  | - |

Abbreviations: MN = malnourished; WN= well nourished; UW=underweight; NOM = non-oedematous malnutrition, OM = oedematous malnutrition; *(WHO)=* Children fulfilling WHOs current diagnostic criteria for severe acute malnutrition; PHA = phytohemaglotinin; DNCB= ninitro cloro-benzene; CMI =cell mediated immunity; PMNC=polymorphnuclear cells; W/H=weight-for-height; H/A= height-for-age; TB= Tuberculosis; * Group of apparently healthy children in the community divided by nutritional status ** compared to themselves after recovery; ↑=higher in malnourished than well-nourished; ↓=lower in malnourished than well-nourished; 0= not different in malnourished and well-nourished; - = not assessed;
