# Supplementary material for: The Immune System in Children with Malnutrition—A Systematic Review
Source: PLoS One. 2014 Aug 25;9(8):e105017. doi: 10.1371/journal.pone.0105017 (PMC4143239; doi:10.1371/journal.pone.0105017)
Supplement: Table S11 — Articles describing lymphocyte subsets in children with malnutrition. (DOCX) [file pone.0105017.s012.docx]

**Table S11: Articles describing lymphocyte subsets in children with malnutrition.**

| **Author, year** | **Country** | **Age, months** | **MN** | **Infections, MN?** | **WN controls** | **Infections, WN?** | **Lymphocytes** | **T-cells** | **CD4-count** | **B-cells** | **Response to PHA** | **Other** | **OM vs. NOM?** |
| --- | --- | --- | --- | --- | --- | --- | --- | --- | --- | --- | --- | --- | --- |
| **Hughes 2009** | Zambia | 12-60 | 56 NOM or OM | yes, 24 HIV | 86 | no | 0 | - | ↑ | - | - | In HIV: drop in CD4 count despite re-nutrition | yes, CD4 count ↑ in OM |
| **Nassar 2009** | Egypt | Mean 11 | 18 NOM, 12 OM | ? | 12 & ** | ? | - | - | - | - | - | More lymphocytes with markers of apoptosis (CD95) in MN | no |
| **Nassar 2007** | Egypt | Mean 12 | 18 NOM  14 OM | ? | 14 | no | - | - | ↑ | - | - |  | yes, CD4 count ↑ in OM |
| **Nájera**  **2007** | Mexico | 7-29 | 3 NOM, 2 OM, 3 UW | yes | 21 | 10 of them | 0 | ↑ | 0 |  | - | T cells ↓ by infection in WN. With infection, effector T cells (CD4+CD62L+) ↓ in MN | ? |
| **Rodríguez 2007** | Mexico | 6-60 | 6 MAM  6 NOM | yes | 12 | yes | - | - | - | - | - | CD4 and CD8 cells with activation markers (CD69 and CD25) ↓ in MN – reversed with leptin | - |
| **Gonzáles 2006** | Mexico | 24-36 | 2 NOM | yes | 2 | yes | - | - | - | - | - | Various genes downregulated in lymphocytes of MN  Control patients younger than study patients | - |
| **Rodríguez 2005** | Mexico | 6-60 | 11 NOM  1 OM | yes | 23 | 12 of them | - | - | - | - | - | Fewer activated T-cells (CD4+ and CD8+ cells with CD69 and CD25) in MN than WN +/- infections | - |
| **El-Hodhod**  **2005** | Egypt | 6-24 | 15 NOM  15 OM | ? | 10 | no | - | - | - | - | - | More cells with markers for apoptosis in MN | no |
| **Vasquez-Garibay 2004** | Mexico | 3-18 | 12 UW/NOM | no | ** | no | - | - | - | - | ↓ |  | - |
| **Nájera 2004** | Mexico | 8-29 | 7 NOM, 3 OM, 5 UW | yes | 22 | 12 of them | 0 | 0 | 0 | ↓ |  | Lymphocyte subsets more affected by infections than by nutritional status | ? |
| **Hagel 2003** | Vene-zuela | Mean 41 | 45 UW | ascaris | 35 | some | - | ↓ | ↓ | ↑↓ |  | B-cells ↑ in mild and mod UW, ↓ in severe UW; CD4+CD45RA ↑, CD4CD45Ro↓, CD4+CD25+↓ | - |
| **Vasquez-Garibay 2002** | Mexico | 3-18 | 12 NOM | (no) | ** | - | - | - | - | - | ↓ |  | - |
| **Nájera 2002** | Mexico | 6 – 29 | 7 NOM, 3 OM, 4 UW | yes | 12 | yes | - | 0 | 0 | ↓ | ↓ | Fewer T-cells with activation marker CD69 in response to PHA | ? |
| **Nájera 2001 (b)** | Mexico | 6-24 | 6 MAM, 6 NOM, 3 OM | yes | 22 | 12 of them | 0 |  |  |  |  | Fewer mature memory T-cells (CD4+CD45RO+) in malnourished infected than well-nourished | ? |
| **Noureldin 1999** | Egypt | Mean 6 & 16 | 8 NOM  6 MK | ? | 100 | ? | - | - | 0 | - | - | Monoclonal antibodies and microscopy | ? |
| **Rikimaru**  **1998** | Ghana | 8-36 | 28 OM, 27 NOM, 39 UW | (no) | 61 | (no) | - | ↑ | 0 | ↓ | - | Higher CRP in malnourished, despite no apparent infections. HLA-DR unchanged. | B-cells , CD8-count ↓ in OM |
| **Ortiz 1995** | Mexico | 6-13 | 6 5 NOM  1 OM | yes | ** | no | - | - | - | - | ↓ |  | - |
| **Chevalier 1994** | Bolivia | 6-55 | 64 NOM or OM | yes | ** | no | - | ↓ | - | - | - | Monoclonal antibodies and microscopy: ↑ immature thymocytes in MN. Thymulin increased CD3 cells and decreased immature cells | ? |
| **Parent 1994** | Bolivia | 6-55 | 13 NOM, 16 OM, 13 MK | yes | 15 | no | - | ↓ | 0 | 0 | - | Monoclonal antibodies and microscopy. ↑ of immature thymocytes in MN. Unclear controls. | no |
| **Ozkan 1993** | Turkey | 3-24 | 29 UW | yes | 15 | yes | - | ↓ | ↓ | - | - | Monoclonal antibodies: CD8 ↓, CD4/CD8 unchanged | - |
| **Fakhir 1989** | India | 6-60 | 22 UW, 54 NOM, 14 MK, 10 OM | (no) | 25 | no | ↓ | ↓ | - | - | - | Lower lymphoctyes, T-cells and small lymphocytes in severely MN than WN |  |
| **Fakhir 1988** | India | ? | 22 UW, 54 NOM,14 MK, 10 OM | no | 25 | no | - | ↓ | - | - | - | No lower in underweight.  Same children as above? | no |
| **Fakhir 1988** | India | 6-60 | 22 UW, 54 NOM,10 OM, 14 MK | (no) | 25 | no | - | - | - | 0/↑ | - | Method ? Absolute B-cell count: 0  B-cell percentage: ↑.Same population as study above | no |
| **Keusch 1987** | Guate-mala | 3-30 | 31 OM *(WHO)* | yes | 41 | no | - | ↓ | - | - | - | Response to stimulation with thymosine in MN | - |
| **Cruz 1987** | Guate-mala | 14-38 | 8 OM *(WHO)* | (yes) | 41 | no | - | ↓ | - | - | - | Still low no. of T-cells after nutritional rehab.  Response to thymosin in lymphocytes of MN | - |
| **Salimonu 1985** | Nigeria | 12-48 | 58 NOM  13 OM | ? | 22 | ? | - | ↓ | - | - | - | T-cells= “E-rosettes”. K<M and MN<WN | ↓ E-rosettes I OM |
| **Joffe 1983** | South Africa | 6-24 | 7 OM *(WHO)* | (no) | 25 | no | - | ↓ | ↓ | - | - | Identified as cells lysed by complement and monoclonal antibodies to CD3, CD4, CD8. Adult controls | - |
| **Murthy 1982** | India | 12-60 | 6 OM *(WHO)* | yes | 5 | no |  |  |  |  | ↓ | Cell-cycle prolonged in lymphocytes of children with OM, | - |
| **Salimonu 1982** | Nigeria | 12-48 | 16 NOM, 29 OM | (no) | 18 | no | - | ↓ | - | 0 | - | Rise in T-cells with tetanus vaccination in NOM, not on WN or OM | ? |
| **McMurray 1981(2)** | Colom-bia | 24 | 8 UW | (no) | 23 | (no) | ↓ | - | - | - | ↓ | Community cohort children followed from 8-104 weeks | - |
| **McMurray 1981 (1)** | Colom-bia | 18-60 | 11 NOM  32 OM | (yes) | 25 | no | 0 | - | - | - | ↓ | Hospital based study | ? |
| **Olusi 1980** | Nigeria | 12-60 | 30 OM *(WHO)* | ? | 30 | ? | 0 | ↓ | - | - | - | Response to thymosin in MN, not in WN | - |
| **Puri 1980** | India | 6-60 | 9 NOM, 8 MK, 5 OM | yes | 13 | yes | - | ↓ | - | 0 | ↓ | Only reduced ”late T-cells” (rosettes after long incubation with RBC) | no |
| **Mahalanabis 1979** | India | ? | 15 OM*(WHO)* | ? | 14 | no | - | ↓ | - | 0 | - | Controls were adults  Higher density of lymphocytes in kwashiorkor | - |
| **Beatty 1978** | South Africa | 10-48 | 12 OM *(WHO)* | (yes) | 10 | no | - | - | - | - | 0/↓ | Decreased response to PHA when lymphocytes were cultivated in serum from MN. | - |
| **Kumar 1978** | India | 12-60 | 27 OM, 8 K, 9 UW | ? | 25 | ? | - | ↓ | - | - |  | Only reduced in NOM and OM, not in UW | no |
| **Kielmann 1977** | India | 0-24 | 13 NOM, 18 UW | no | 15 | no | - | - | - | - | 0/↓ | Only reduced in NOM, not in UW | - |
| **Moore 1977** | Gambia | 13-29 | 7 NOM,5 MK,4 OM | yes | 16 | no | - | - | - | - | 0 | No difference when cultured in calf serum  Reduced response In autologous plasma | no |
| **Keusch 1977** | Guate-mala | Mean 36 | 5 OM, 7 MK, 2 NOM | yes | 3 | yes | 0 | ↓ | - | - | - | EM. Frequent plasmacytoid cells in blood | - |
| **Smith 1977** | Tunisia | 3 – 18 | 39 NOM, 29 MK | yes | 58 | no | - | ↓ | - | - | - |  | - |
| **Kulapongs 1977** | Thailand | 12-60 | 24 NOM or OM | yes | 10 | ? | - | ↓ | - | 0 | ↓ |  | - |
| **Schlesinger 1977** | Chile | 3-18 | 22 NOM | some | 60 | some | - | - | - | - | 0 |  | - |
| **Reddy 1976** | India | 12-60 | 15 NOM or OM, 9 UW | ? | 15 | ? | 0 | ↓ | - | - | ↓ |  | - |
| **Nahani 1976** | Iran | 4-38 | 24 NOM,  29 UW* | No | 35* | no | - | ↓ | - | 0 | - | Percentage of T and B cells. Absolute values not given | - |
| **Schopfer 1976** | Cote d’Ivoire | ? | 46 OM *(WHO)* | (no) | 40 | ? | ↑ | - | - | - | - | Higher eosinophils | - |
| **Schopfer 1976** | Cote d’Ivoire | ? | 27 OM *(WHO)* | (no) | 28 | no | 0 | ↓ | - | - | ↓ | ↑ neutrophils, eosinophils, atypical lymphocytes and plasmacytoid cells in MN | - |
| **Schopfer 1976** | Cote d’Ivoire | ? | 72 OM *(WHO)* | (no) | 36 | no | 0 | - | - | - | - | ↑ neutrophiles. Pleomorphism of granulae. Immature PMNC, plasmacytoid lymphocytes. ↓ eosinophils | - |
| **Purtilo 1976** | Brazil | 15-50 | 3 OM, 5 NOM, 41 UW* | yes | 14 | yes | 0 | - | - | - | - |  | no |
| **Rosen 1975** | South Africa | 6-24 | 35 OM *(WHO)* | some | 21 | some | ↓ | - | - | - | - |  | - |
| **Rabson 1975** | South Africa | 6-24 | 15 OM *(WHO)* | ? | 10 | some | - | 0 | - | - | - |  | - |
| **Bang 1975** | India | 4-60 | 17 OM, 5 NOM | yes | 7 | no | - | ↓ | - | 0 | - | Rosetting methods. Adult controls | Lowest values in kwashorkor |
| **Moore 1974** | Gambia | 9-36 | 9 OM, 7 NOM | (yes) | 18 | (no) | - | - | - | - | 0 |  | no |
| **Bhaskaram 1974** | India | 12-60 | 8UW,14 OM  8 NOM | yes/  no | 11 | no | 0 | ↓ | - | - | ↓ | Only↓ in severe MN  No difference in UW from WN | no |
| **Schlesinger 1974** | Chile | 3-18 | 13 NOM | (yes) | 8 | (yes) | - | - | - | - | 0 |  | - |
| **Ferguson 1974** | Ghana | 12-39 | 7 OM  3 NOM | ? | 10 | ? | - | ↓ | - | - | 0 | Tendency to lower PHA response in MN | ? |
| **Grace 1972** | South Africa | 3-60 | 30 ? | ? | 10 | no | - | - | - | - | ↓ |  | - |
| **Geefhuysen 1971** | South Africa | 6-30 | 18 OM *(WHO)* | 13 of them | 9 | yes | - | - | - | - | ↓ |  | - |
| **Smythe 1971** | South Africa | 3-60 | 14 ? | ? | 9 | ? | - | - | - | - | ↓ |  | - |

Legend: MN= malnourished; WN= well-nourished; NOM= non-oedematous malnutrition; OM= oedematous malnutrition; MK= marasmic kwashiorkor, UW=Underweight, defined by low weight-for-age; *(WHO)=* Children fulfilling WHOs current diagnostic criteria for severe acute malnutrition; PHA= phytohemaglutinin;↓=lower in malnourished than well-nourished; ↑=higher in malnourished than well-nourished; 0= not different in malnourished and well-nourished; E-rosettes= erythrocyte rosettes, older method to quantify T-cells; EAC-rosettes= older method to quantify B-cells; HMS=hexose monophosphate shunt
