# Supplementary material for: The Immune System in Children with Malnutrition—A Systematic Review
Source: PLoS One. 2014 Aug 25;9(8):e105017. doi: 10.1371/journal.pone.0105017 (PMC4143239; doi:10.1371/journal.pone.0105017)
Supplement: Table S12 — Articles describing antibody levels in children with malnutrition. (DOCX) [file pone.0105017.s013.docx]

**Table S12: Articles describing antibody levels in children with malnutrition.**

| **Author, year** | **Country** | **Age, months** | **No MN** | **Infections, MN?** | **No WN controls** | **Infections, WN?** | **IgG** | **IgM** | **IgA** | **IgE** | **IgD** | **Other** | **Comments** | **OM vs NOM?** |
| --- | --- | --- | --- | --- | --- | --- | --- | --- | --- | --- | --- | --- | --- | --- |
| **Cripps 2008** | Papua New Guinea | 1-60 | *99 UW | yes | *  129 | yes | 0 | 0 | 0 | - | - | - |  | - |
| **Hagel 2003** | Venezuela | Mean 41 | 45 UW | yes | 35 | some | - | - | - | ↑↓ | - | - | IgE ↑ in moderate MN, ↓in severe MN | - |
| **Forte 2003** | Brazil | 24-48 | 18 UW | no | 15 | no | - | - | - | ↓ | - | - |  | - |
| **Rikimaru 1998** | Ghana | 8-36 | 49 UW,28 OM, 27 NOM | (no) | 61 | no | 0 | 0 | ↑ | - | - | - |  | 0 |
| **Ozkan 1993** | Turkey | 3-24 | 29 UW | yes | 15 | yes | ↑ | ↑ | ↑ | - | - | - |  | - |
| **Fakhir 1988** | India | 6-60 | 22 UW, 54 NOM, 14 MK, 10 OM | (no) | 25 | no | ↑ | ↑ | ↑ | - | - | - | IgA ↑ in NOM and OM than in UW | no |
| **Taddese 1988** | Ethiopia | 18-30 | 20 OM *(WHO)* | (no) | 40 | no | ↓ | (↓) | (↑) | - | - | - |  | - |
| **Salimonu 1985** | Nigeria | 12-48 | 58 NOM, 13 OM | ? | 22 | ? | - | - | - | - | - | immune complexes: 0 |  | 0 |
| **Beatty 1983** | South Africa |  | 4 OM *(WHO)* | Yes | ** | no | (↓) | (↓) | (↑) | - | - | - |  | - |
| **McMurray 1981** | Colombia | 18-60 | 21 OM, 11 MK, 11 NOM | Yes | 25 | no | OM: ↓  NOM 0 MK:0 | OM:0  NOM ↑ MK:↑ | OM:↑  NOM:0 | - | OM: ↑  NOM:0 MK:0 | - |  | yes |
| **Puri 1980** | India | 6-60 | 9 NOM, 5 OM, 8 MK | Yes | 13 | yes | 0 | 0 | - | - | - | - |  | ? |
| **Watson 1978** | Colombia | 24-60 | *26 UW | No | *27 | no | 0 | 0 | ↑ | - | - | - |  | - |
| **Ibrahim 1978** | Egypt | 6-36 | 37 NOM, 18 OM, 15 MK | (no) | 15 | no | - | - | ↑/0 | - | - | - | Only ↑ in OM | Yes, IgA ↑ in OM |
| **Beatty 1978** | South Africa | 10-48 | 12 OM *(WHO)* | minor | 10 | no | (↑) | ↑ | ↑ | ↑ | - | - |  | - |
| **McMurray 1977** | Colombia | 18-24 | *12 UW | (no) | 27 |  | 0 | 0 | ↑ | - | - | - |  | - |
| **Awdeh 1977** | Lebanon | 3-12 | 10 NOM, 11 UW | ? | 6 | ? | 0 | 0 | 0 | - | - | - |  | - |
| **Kielmann 1977** | India | 0-24 | 15 NOM, 19 UW* | no | 19 | no | 0 | 0 | ↓ | - | - | - |  | - |
| **Suskind 1977** | Thailand | 24-60 | 14 NOM, 7 MK, 7 OM | yes | 18 | yes | 0 | 0 | ↑ | ↑ | - | - |  | IgA and IgD ↑ in OM |
| **Suskind 1976** | Thailand | 24-60 | 14 OM, 14 NOM | yes | 30 | 18 | 0 | 0 | 0/↑ | ↑ | ↑ | - | IgD and IgA highest with oedema | Yes, IgD, IgA ↑ in OM |
| **Nahani 1976** | Iran | 4-38 | 24 NOM, 29 UW* | (no) | 35 | no | 0 | 0 | 0 | - | ↑ | - |  | - |
| **Purtilo 1976** | Brazil | 15-50 | *3 OM, 5 NOM, 41 UW | yes | 14* | (no) | 0 | 0 | 0 | 0 | 0 | - | Correlated with parasite burden | - |
| **Sirisinha 1975** | Thailand | 12-60 | 8 OM, 7 NOM, 9 UW | yes | 23 | no | - | - | ↑ | - | - | - |  | ? |
| **Olusi 1975** | Nigeria | 12-60 | 50 OM, 25 NOM | ? | 35 | yes | 0 | - | 0 | - | - | - |  | - |
| **Casazza 1972** | Indonesia | 6-36 | 118 UW | ? | 95 | ? | 0 | ↑ | ↑ | - | - | - |  | - |
| **Rosen 1971** | South Africa | 5-60 | 30 OM, 4 NOM, 16 MK | 41 | 14 | 7 | 0 | 0 | ↑ | - | - | - |  | Yes, IgG, IgA ↑in NOM |
| **Watson 1970** | South Africa | 7-34 | 25 OM *(WHO)* | yes | 12 | yes | 0 | 0 | ↑ | - | - | - | - | - |
| **Aref 1970** | Egypt | 3-64 | 27 OM *(WHO)* | yes | 24 | no | ↓  ↑ | ↓ | ↓  ↑ | - | - | - | ↓ in <1 year, ↑ in >1 year | - |
| **El-Ghomly 1970** | Egypt | 10-36 | 21 OM *(WHO)* | (no) | ? | ? | 0 | (↓) | ↑ | - | - | - | Higher IgA with dermatosis | (yes) |
| **El-Ghomly 1970** | Egypt | 12-30 | 17 NOM | (no) | 15 | ? | 0 | ↑ | 0 | - | - | - | Splenomegali frequent | - |
| **Keet 1969** | South Africa | 7 - 34 | 11 OM *(WHO)* | yes | 11 | yes | 0 | 0 | ↑ | - | - | - |  | - |
| **Najjar 1969** | Lebanon | 3-30 | 16 NOM | yes | 23 | no | 0 | ↑ | ↑ | - | - | - |  | - |
| **Cohen 1962** | South Africa | 9-22 | 7 OM *(WHO)* | yes | ** | (no) | - | - | - | - | - | y-globulin: 0 | y-globulin metabolism 0 | - |

Legend: MN= malnourished; WN= well-nourished; NOM= non-oedematous malnutrition; OM= oedematous malnutrition; MK= marasmic kwashiorkor, UW= underweight, defined by low weight-for-age; *(WHO)=*Children fulfilling WHOs current diagnostic criteria for severe acute malnutrition; IgA = immunoglobulin A; IgM = immunoglobulin M; IgG = immunoglobulin G; IgE= Immunoglobulin E, IgD = immunoglobulin D ; ↑= higher in malnourished children than well-nourished; ↓ = lower in malnourished than well-nourished, 0 = not different in malnourished and well-nourished; *= group of children compared according to nutritional status; **=malnourished children compared to themselves after nutritional recovery; - = not assessed; ? = not statted
