# Supplementary material for: The Immune System in Children with Malnutrition—A Systematic Review
Source: PLoS One. 2014 Aug 25;9(8):e105017. doi: 10.1371/journal.pone.0105017 (PMC4143239; doi:10.1371/journal.pone.0105017)
Supplement: Table S13 — Articles describing humoral vaccination responses in children with malnutrition. (DOCX) [file pone.0105017.s014.docx]

**Table S13: Articles describing humoral vaccination responses in children with malnutrition.**

| **Author, year** | **Country** | **Age, months** | **No MN** | **Infections, MN?** | **No WN con-trols** | **Infections, WN?** | **Acceptable seroconversion?** | **Titre response** | **Vaccination**  **Antigen** | **Specific antibodies** | **Comments** | **NOM vs OM?** |
| --- | --- | --- | --- | --- | --- | --- | --- | --- | --- | --- | --- | --- |
| **Asturias 2009** | Guatemala | 2-12 | 89 mod UW | No | 325 | no | Yes | 0 | HepB |  |  | - |
| **Fillol 2009** | Senegal | 2-59 | 142 stu., 19 moderate wast. | (no) | 161 | (no) | - | - | - | Malaria IgG:  stun↓,wast 0 |  | - |
| **Cripps 2008** | Papua New Guinea | 1-60 | 99 UW | yes | 129 | yes | - | - | - | *H.infl, E.coli*. IgG: 0 sIgA : 0 | Lower in some age-groups, not overall | - |
| **Hagel 2003** | Venezuela | Mean 41 | 45 UW | yes | 20 | yes | - | - | - | *Ascaris* IgE: ↓ | All infected with Ascaris | - |
| **Waibale 1999** | Uganda | 15-42 | 243 total* | ? | * | ? | - | ↓ | Mea | - | Low titres associated with stunting, not wasting on examination. Nutritional status not measured at vaccination. | - |
| **El-Gamal 1996** | Egypt | Mean 13,6 | 12 OM, 19 NOM | ? | 13 | ? | Yes | ↓ | HepB | - | 87% sero-converted | Lower in OM |
| **Brussow 1995** | Ecuador | 12-59 | 215 UW* | ? | 507* | ? | - | - | - | UW: ↓ titres to RSV , 0 to RV, *E.coli*, LPS | Not SAM | - |
| **Dao 1992** | Mali | 8-22 | 36 Stu  60 moderate wast* | No | 104 | no | yes: 0 | - | Mea | - | Not severe MN | - |
| **Chopra 1989** | India | 1-12 | 20 UW* | No | 42* | no | yes: 0 | - | POL | - | Not severe MN | - |
| **Smedman 1988** | Guinea Bissau | 8-19 | 63 UW* | ? | 99 | ? | yes: 0 | - | Mea | - | Not severe MN | - |
| **Smedman 1986** | Guinea Bissau | 8-19 | 132 total* | ? | * | ? | yes: 0 | 0 | Mea | - | Only 5 children severely UW, 2 of these did not seroconvert | - |
| **Bhaskaram 1986** | India | 9-36 | 14 UW | ? | 6 | ? | yes: 0 | - | Mea | - | Notes lower general morbidity in 12 weeks after vaccination than in unvaccinated children | - |
| **Green-wood 1986** | Nigeria | 4-24 | Total 126* | no | total 126* | no | yes: 0 | 0 | DTP, POL, Mea, Men, TPH | - | UW, not severe MN | - |
| **Baer 1986** | Trinidad | 12-36 | 29 mild wasting | No | 22 | no | yes: 0 | 0 | Mea | - | Not severe MN | - |
| **Halsey 1985** | Haiti | 6-12 | Total 333* | ? | * | ? | yes: 0 | - | Mea | - | Mild/mod UW | - |
| **Ekunwe 1985** | Nigeria | 6-24 | 35 UW* | No | 73 | no | yes: 0 | - | Mea | - | Mild/mod MN | - |
| **Idris 1983** | Sudan | 13-36 | 23 NOM  12 MK | ? | 35 | ? | yes: 0 | ↓ | Mea | - | Reactions: Fever, pneumonia, diarrhoea: ↑ Rash ↓ | titres ↓in K |
| **Salimonu 1982** | Nigeria | 12-48 | 31 OM, 20 NOM, 9 MK | No | 18 | no | yes: 0 | 0,↓ | TTA, Mea (only K) | - |  | no for TTA |
| **Powell 1982** | Zimbabwe | Mean 25 | 12 OM *(WHO)* | (no) | 11 | no | no: only 2/12 | ↓ | Mea | - | No severe reactions | - |
| **Greenwood 1980** | Nigeria | 0-60 | 31 UW* | No | 16 | no | Yes | - | Men | - | Not severe MN |  |
| **Paul 1979** | India | 0-36 | 37 UW* | ? | 40* | ? | yes:(Diph)0 no (TTA) only 25/37 | ↓,↓ | Diph, TTA | - |  | - |
| **Wesley 1979** | South Africa | 6-24 | 20 low MUAC | ? | 26 | no | no: ↓ |  | Mea | - | Response delayed: By day 10 only 15% seroconverted, by day 21, 70%  More vaccination reaction in MN | - |
| **McMurray 1979** | Colombia | 10 | 35 UW* | No | 39* | no | yes: 0 | 0 | Mea | - | No difference in adverse reactions | - |
| **Kielmann 1977** | India | 0-24 | ? | No | ? | no | - | 0 | TTA | - |  | - |
| **Suskind 1977** | Thailand | 24-60 | 2 NOM, 3 MK, 5 OM | yes | 10 | no | - | ↓ | TPH | - | Response delayed in MN | - |
| **Hafez 1977** | Egypt | 11-23 | 45 OM, 38 NOM | ? | 30 | ? | no: ↓ | ↓  ↓  ↓ | Mea  Pol  Diph | - |  | 0 |
| **Awdeh 1977** | Lebanon | 3-12 | 10 NOM, 11 UW | ? | 6 | ? | yes: 0 | 0/↑ | TTA, diph given 3 times | - |  | - |
| **Reddy 1976** | India | 12-60 | 15 NOM or OM, 9 UW | ? | 15 | ? | no (TPH), yes (Diph, TTA) | ↓, 0, 0 | TPH, Diph, TTA | - | TPH only ↓ in severely MN | - |
| **Suskind 1976** | Thailand | 24-60 | 14 OM, 14 NOM | yes | 30 | 18 | - | ↓ | TPH | - |  | yes |
| **El-Molla 1973** | Egypt | 12-24 | 8 OM,7 NOM | ? | 11 | ? | - | ↓/0 | Cole | - | Vibriocidal antibodies: 0 Agglutinating antibodies ↓ | - |
| **Brown 1966** | Uganda | 4-42 | 18 mild UW | No | 36* | no | Yes | 0 | Smallpox | - | Only mild malnutrition | - |
| **Brown 1866** | Uganda | 18-42 | 8 OM *(WHO)* | yes | 6 | (no) | no ↓ | -  - | Yellow fever | - |  | - |
| **Brown 1965** | Uganda | 15-42 | 8 OM *(WHO)* | yes | 6 | yes | no ↓ | - | Yellow fever | - |  | - |
| **Pretorius 1962** | South Africa | ? | 30 OM *(WHO)* | yes | 15 | no | Yes | (↓) | TPH | - | Most responded normally | - |
| **Kahn 1957** | South Africa | 8-36 | 14 OM *(WHO)* | ? | 11 | ? | - | - | - | Iso-haemaglutinin titres: 0 |  | - |

Legend: MN= malnourished; WN= well-nourished; NOM= non-oedematous malnutrition; OM= oedematous malnutrition; UW=Underweight, defined by low weight/age; Stu=stunted, defined by low height-for-age; wast = wasting, defined by low weight-for-height; *(WHO)=*Children fulfilling WHOs current diagnostic criteria for severe acute malnutrition; PPS= pneumococcal polysaccharide, KLA= keyhole limpit antigen, TTA= tetanus toxoid antigen. DTP= triple vaccine (diphtheria, tetanus, pertussis); TPH=typhoid antigen; HepB= hepatitis B recombinant vaccine; Mea=measles vaccine; Men=meningococcal group C polysaccharide vaccine; spox= smallpox; Diph= diphtheria; Pol= polio; Cole= Cholera; RSV= respiratory Syncytial Virus; RV= Rota Virus, E. Coli; LPS= lipopolysaccharide; *= population of children divided by nutritional status; ↑=higher in malnourished than well-nourished; ↓=lower in malnourished than well-nourished; 0= not different in malnourished and well-nourished; - = not assessed; ? = not stated
