# Supplementary material for: The Immune System in Children with Malnutrition—A Systematic Review
Source: PLoS One. 2014 Aug 25;9(8):e105017. doi: 10.1371/journal.pone.0105017 (PMC4143239; doi:10.1371/journal.pone.0105017)
Supplement: Table S14 — Articles describing cytokines in malnourished children. (DOCX) [file pone.0105017.s015.docx]

**Table S14: Articles describing cytokines in malnourished children**

| **Author, Year** | **Country** | **Age** | **Malnourished** | **Infections?** | **WN** | **Infections, WN?** | **Assay** | **L-MIF** | **IL-1** | **IL-2** | **IL-4** | **IL-6** | **IL-8** | **IL -10** | **IL- 12** | **IL -18** | **IL-21** | **TNFα** | **IFN γ** | **GM-CSF** | **ER-IS** | **Other** | **OM vs NOM?** |
| --- | --- | --- | --- | --- | --- | --- | --- | --- | --- | --- | --- | --- | --- | --- | --- | --- | --- | --- | --- | --- | --- | --- | --- |
| **Bartz 2014** | Uganda | 6 - 60 | 42 OM  32 NOM  *(WHO)* | yes | ** 14 | no | Plasma, immunoassay |  |  |  |  | 0 | 0 | ↑ |  |  |  | 0 | ↑ | 0 |  | IL1β: 0  Leptin ↓  IGF-1 ↓  Insulin ↓  GH: ↑  Cortisol ↑  Ghelin: ↑ | **?** |
| **Gonzales-Torres 2013** | Mexico | Mean 20,4-24,2 | 10 MAM  7 NOM | yes | 10 | yes | RNA , Intracellular  Plasma |  |  | ↓ |  |  |  |  | ↓ | ↓ | ↓ |  | ↓ |  |  |  | **-** |
| **Hughes 2009** | Zambia | 12-60 | 57 OM, 24 NOM | yes | ** | no | Production in cultivated dendritic cells  Plasma |  |  |  |  |  |  | 0 | ↓ |  |  | 0 | ↓ |  |  |  | **?** |
| **Gonzales-Martinez 2008** | Mexico | 6-60 | 3 MAM  8 NOM or OM | yes | 14 | yes | mRNA |  |  | ↓ | ↑ | ↓ |  | ↑ |  |  |  | ↑ | ↓ |  |  |  | **-** |
| **Rodrigues 2007** | Mexico | 6-48 | 7 NOM  5 MAM | yes | 12 | yes | Intracellular in T cells, flow cytometry |  |  | ↓ |  |  |  | ↑ |  |  |  |  | ↓ |  |  | improved with leptin | **-** |
| **Abo-Shousha 2005** | Egypt | 4-26 | 11 NOM  20 OM  15 UW | yes | 10, | yes, minor | Production in cultivated PBMC |  |  |  |  | ↑ | ↑ |  |  |  |  |  |  | 0/↓ |  | ↓ response to LPS | IL-6, IL-8, GM-CSF ↓ in OM |
| **Rodrigues 2005** | Mexico | 6-60 | 11 NOM  1 OM | yes | 23 | half | Intracellular in cultivated PBMC, flow cytometry |  |  | ↓ |  |  |  | ↑ |  |  |  |  | ↓ |  |  |  | **-** |
| **Manary 2004** | Malawi | 12-60 | 42 NOM | 25 | 13 | yes | Serum, immunoassay |  |  |  |  | ↑ |  |  |  |  |  | 0 |  |  |  | IL-1β ↑ in MN with inf. | **-** |
| **Hagel 2003** | Vene-zuela | Mean 40 | 45 UW* | Ascaris | 20 | Ascaris | Serum, immunoassay |  |  |  | ↑↓ |  |  |  |  |  |  |  |  |  |  | IL-4 ↑ in mod. UW, ↓ in sev. UW | **-** |
| **Hemalatha 2002** | India | 1-60 | Total 120* | men | * | men | CSF, immunoassay |  |  |  |  |  |  |  |  |  |  | 0 |  |  |  |  | **-** |
| **Dulger 2002** | Tyrkey | 12-60 | 10 NOM  15 OM | ? | 18 | no | Serum, immunoassay |  |  |  |  | ↑ |  |  |  |  |  | 0 |  |  |  |  | IL6 tend ↑ in OM |
| **Reid 2002** | Jamaica | Mean 10,1 - 11,4 | 9 NOM  14 OM | yes | ** | no | Serum, immunoassay |  |  |  |  | ↑ |  |  |  |  |  |  |  |  |  |  | (tend to ↑ in OM) |
| **Palacio 2002** | Chile | Mean 14,6 | 15 mod UW | No | 16 | no | Cultivated PBMC, immunoassay |  | 0 |  | ↑ | 0 |  |  |  |  |  | 0 | 0 |  |  | in response to LPS  Tend to ↓ IL-1 | - |
| **Iputo 2002** | South Africa | Mean 15,6 | 21 OM *(WHO)* | no | 11 | no | Plasma |  |  |  |  |  |  |  |  |  |  |  |  |  |  | PGE2 ↑ in K | - |
| **Solis 2002** | Morocco | 6-24 | 22 NOM | ? | ** | ? | Cultivated PBMC, immunoassay |  |  |  |  |  |  |  |  |  |  |  | ↓ |  |  |  | - |
| **Giovambatista 2000** | Argentina | 8-60 | 9 mod. UW | no | 9 | no | Serum, cultured leucocytes,  Immunoassay |  |  |  |  |  |  |  |  |  |  | ↑ |  |  |  |  | - |
| **Fongwo 1999** | Nigeria | ? | 41 NOM  19 OM, 5 MK | ? | 35 | ? | Leukocytes, bioassay | ↓ |  |  |  |  |  |  |  |  |  |  |  |  |  | L-MIF ↓ in both M and K, most in K | Yes |
| **Lotfy 1998** | Egypt | 5-20 | 12 NOM,  12 MK  16 OM | ? | 20 | no | Cultivated PBMC, immunoassay |  | 0/↓ | 0/↓ |  |  |  |  |  |  |  |  |  |  |  | Only ↓ in K | Yes |
| **Malavé 1998** | Vene-zuela | 6-60 | 46 UW | half | 61 | half | Plasma,  Immunoassay |  |  |  |  | 0 |  |  |  |  |  |  |  |  |  |  | - |
| **Malavé 1998** | Vene-zuela | 6-60 | 44 UW | no | 38 | no | Cultivated PBMC , Bioassay |  |  |  |  | ↑ |  |  |  |  |  |  |  |  |  |  | - |
| **Sauerwein 1997** | Kenya | mean 29-37 | 30 OM  16 NOM | no | 39 | no | Plasma, immunoassay |  |  |  |  | ↑ |  |  |  |  |  |  |  |  |  | sTNFrp55↑  sTNFr-p75↑  IL1-Ra and sIL6-R: 0 | Yes ↑in K |
| **Aslan 1996** | Turkey | mean 19 | 13 OM  15 NOM | yes | 12 | yes | Plasma, immunoassay |  | ↓ |  |  |  |  |  |  |  |  |  |  | ↓ |  |  | Yes ↓in OM |
| **Doherty 1994** | Jam-aica | 9 - 20 | 2 UW,  5 NOM, 5 OM, 4 MK | ? | ** | no | Whole blood w LPS, bioassay |  |  |  |  | 0/↓ |  |  |  |  |  | 0/↓ |  |  |  | unstimulated: 0, response to LPS ↓ | no |
| **Mayapatec 1993** | Nige-ria | Median 19 | 12 OM  24 NOM | ? | 12 | no | Whole blood, urine |  |  |  |  |  |  |  |  |  |  |  |  |  |  | LTB4↓ n OM  LTC4, LTE4 ↑ in OM | yes, NOM like WN |
| **Bhaskaram 1986** | India | 24-60 | 5 OM  6 NOM | no | ? | ? | Cultivated macrophages, bioassay |  | ↓ |  |  |  |  |  |  |  |  |  |  |  |  |  | yes ↓in OM |
| **Salimonu 1982** | Nige-ria | 12-60 | 14 NOM  5 OM | no | 16 | no | Plasma, bioassay |  |  |  |  |  |  |  |  |  |  |  |  |  |  | IFN: 0 | **-** |
| **Salimonu 1982** | Nige-ria | ? | 15 NOM, 22 OM,  2 MK | no | 42 | no | Serum, bioassay |  |  |  |  |  |  |  |  |  |  |  |  |  | ↑ |  | yes, ↑  in K |
| **Heresi 1981** | Chile | 6-18 | 14 NOM | no | 27 | no | Leucocytes, bioassay | 0 |  |  |  |  |  |  |  |  |  |  |  |  |  |  | **-** |
| **Kobielowa 1979** | Po-land | 3-21 | 30 UW | yes | 21 | no | Serum, bioassay |  |  |  |  |  |  |  |  |  |  |  |  |  |  | ↑ lympho-cytotoxin activity | **-** |
| **Beatty 1979** | South Africa | 12-50 | 17 OM *(WHO)* | ? | adults | no | Serum, bioassay |  | ↓ |  |  |  |  |  |  |  |  |  |  |  |  |  | **-** |
| **Beatty 1978** | South Africa | 10-48 | 11 OM  1 MK | minor | 10 | no | Serum, bioassay |  | 0/↓ |  |  |  |  |  |  |  |  |  |  |  |  |  | **-** |
| **Moore 1977** | Gam-bia | 13-29 | 7 NOM, 4 OM  5 MK | some | 16 | yes | Plasma, bioassay |  | 0/↓ |  |  |  |  |  |  |  |  |  |  |  |  |  | **No** |
| **Schlesinger 1977** | Chile | 3-18 | 22 NOM | some | 60 | some | Leucocytes, bioassay |  |  |  |  |  |  |  |  |  |  |  | ↓ |  |  |  | **-** |
| **Lomnitzer 1976** | South Africa | 6-24 | 25 OM *(WHO)* | half | 12 | half | Leucocytes, bioassay | ↓ |  |  |  |  |  |  |  |  |  |  |  |  |  |  | **-** |
| **Heyworth 1975** | Gambia | 9-52 | 4 OM, 7 NOM  7 MK, 1 UW | some | 4 | no | Plasma, bioassay |  | 0/↓ |  |  |  |  |  |  |  |  |  |  |  |  | plasma inhibited response to PHA. | **-** |

Legend: MN= malnourished; WN= well-nourished; UW= under-weight, defined by low weight-for-age; OM: oedematous malnutrition; NOM= non-oedematous malnutrition; MK=marasmic kwashiorkor, defined by both wasting and oedema; Stu=stunted, defined by low height-for-age; MAM=moderate acute malnutrition, defined by low weight-for-height; mod= moderate; *(WHO)=* Children fulfilling WHOs current diagnostic criteria for severe acute malnutrition; susp.= suspected; *= population of children divided by nutritional status, **malnourished children compared to themselves after nutritional recovery; L-MIF= Leucocyte migration Inhibition Factor; IL= Interleukin; TNFα= Tumor Necrosis Factor Alpha, IFNγ= Interferon Gamma, GM-CSF= Granulocyte Macrophage-Colony Stimulation Factor, ER-IS= Erythrocyte-rosette Inhibiting Substance, TB= Tuberculosis; men= meningitis; LPS=Lipopolysaccharide; IL1-Ra= Inteleukin-1-recepto antagonist; sIL6-R = soluble Interleukin-6 receptor; sTNFr-p55 = soluble Tumor Necrosis Factor receptor-p55; sTNFr-p75= soluble Tumor Necrosis Factor receptor-p75; PBMC= Peripheral Blood Mononuclear cells; IGF-1: Insulin-like growth factor 1, GH = Growth hormone; PGE = Prostaglandin; LTB4= leukotriene B4; LTC4= leukotriene C4; LTE4= leukotriene E4; PHA= phyto-hemaglutininin
